# Supplementary material for: Dynamics of individual molecular shuttles under mechanical force
Source: Nat Commun. 2018 Oct 30;9:4512. doi: 10.1038/s41467-018-06905-8 (PMC6207653; doi:10.1038/s41467-018-06905-8)
Supplement: Supplementary file 1 — Supplementary Information [file 41467_2018_6905_MOESM1_ESM.pdf]

Supplementary Information

Dynamics of individual molecular shuttles under mechanical force

Teresa Naranjo, Kateryna M. Lemishko, Sara de Lorenzo, Álvaro Somoza, Félix Ritort, Emilio M. Pérez and Borja Ibarra.

Correspondence to: emilio.perez@imdea.org borja.ibarra@imdea.org

S1 Supplementary Methods
S2 Supplementary Figures

S1 Supplementary Methods

S1.1 Chemical synthesis of the molecular shuttle

The chemical structure of the shuttle comprises a strong binding station, namely fumaramide (green in Fig. 1a), which is known to be the best fit for a tetraamide macrocycle, because its two amide carbonyls are held by the *trans* double bond in the correct geometry to form four strong hydrogen bonds with the macrocycle. Separated by an oligoethyleneglycol spacer of approximately 15 nm in length (see below) we placed a succinic amide-ester station, (orange in Fig. 1a). This station shows less affinity for the macrocycle than the fumaramide station, due to its flexibility and the substitution of one of the amides with an ester, which is a significantly weaker H-bond acceptor. At the ends of the polar spacer two bulky diphenylethyl groups serve as stoppers to prevent unthreading of the macrocycle. One of the stopper ends is branched to include a biotin group. The synthesis of the axle for the shuttle was carried out in 12 steps (see below). Macrocyclization around the fumaramide station was performed using a purposely synthesized U-shape (compound 14) The macrocycle contains an azide-decorated isophthalic acid chloride derivative (compound 13) at a single position for the covalent attachment of a polydT oligonucleotide.

General. All solvents were dried according to standard procedures. Reagents were used as purchased. All air-sensitive reactions were carried out under argon atmosphere. Flash chromatography was performed using silica gel (Merck, Kieselgel 60, 230-240 mesh, or Scharlau 60, 230-240 mesh). Analytical thin layer chromatographies (TLC) were performed using aluminium-coated Merck Kieselgel 60 F254 plates. NMR spectra were recorded on a BrukerAvance 400 (^{1}H: 400 MHz; ^{13}C: 100 MHz; COSY: 400 MHz; HSQC: 400 MHz), spectrometer at 298 K, unless otherwise stated, using partially deuterated solvents as internal standards. Coupling constants (J) are denoted in Hz and chemical shifts (δ) in ppm. Multiplicities are denoted as follows: s = singlet, d = doublet, t = triplet, m = multiplet, br = broad. Fast Atom Bombardment (FAB) and Matrix-assisted Laser desorption ionization (coupled to a Time-Of-Flight analyzer) experiments (MALDI-TOF) were recorded on a VS AutoSpec spectrometer and a Bruker ULTRAFLEX III spectrometer, respectively.

Scheme of the rotaxane synthesis

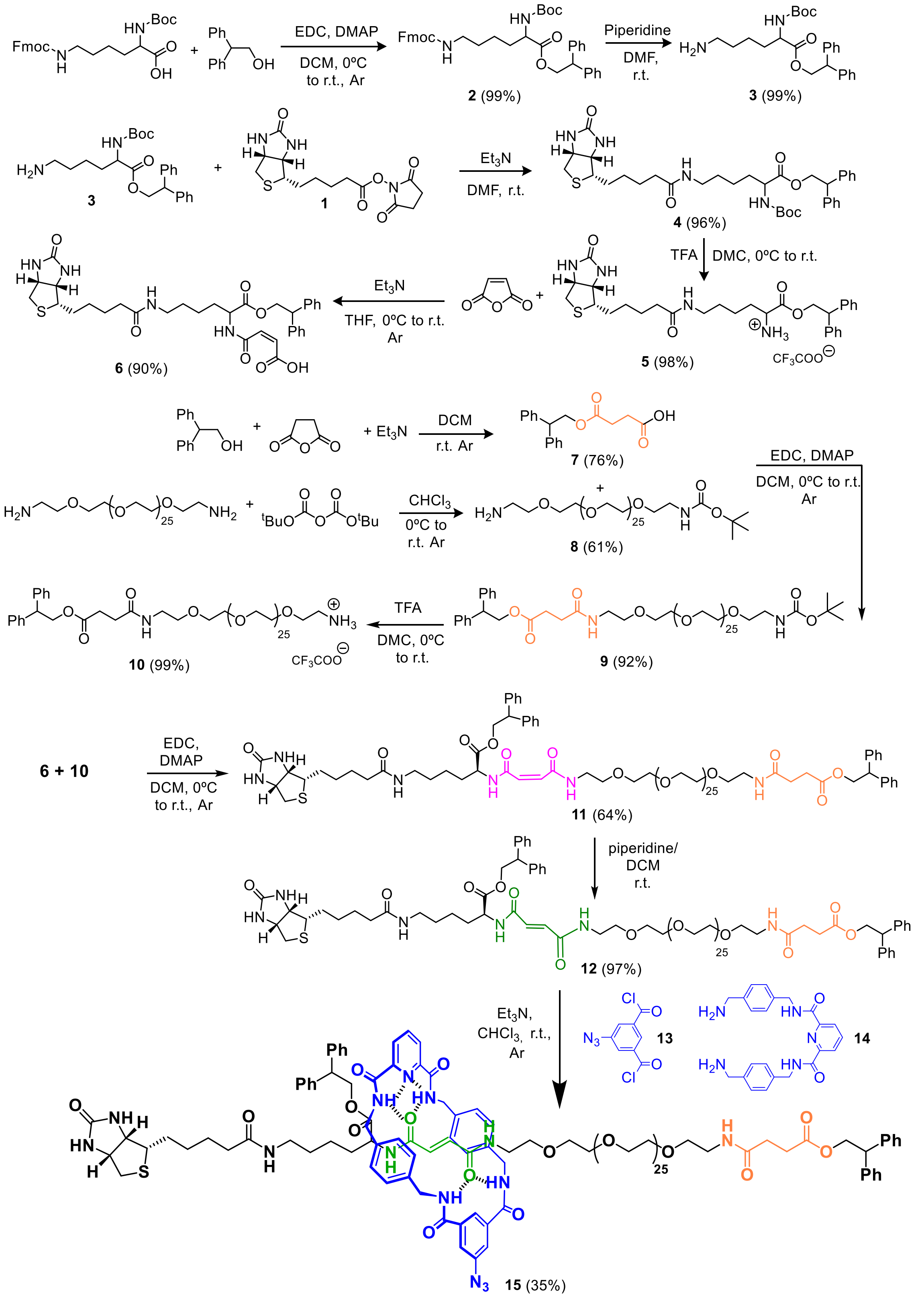

<u>*N*-Hydroxysuccinimidobiotin</u>

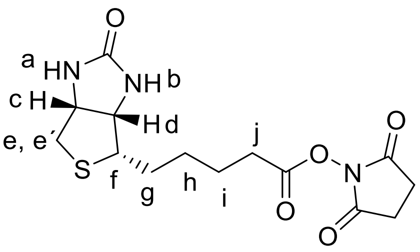

Compound 1

To a solution of D-biotin (75 mg, 0.31 mmol) and *N*-hydroxysuccinimide (39 mg, 0.34 mmol) in DMF, 1-ethyl-3-(3-dimethylaminopropyl)carbodiimide hydrochloride (EDCI) was added (71 mg, 0.37 mmol). After being stirred for 24h at room temperature, the reaction solution was concentrated to obtain a white solid. The white solid was washed by methanol several times to furnish compound **1** in a 90% yield (96mg, 0.28mmol). ^{1}H NMR ([D6]dimethyl sulfoxide): δ = 6.41 (s, 1H, H_a), 6.35 (s, 1H, H_b), 4.30 (m, 1H, H_c), 4.15 (m, 1H, H_d), 3.10 (m, 1H, H_f), 2.83 (dd, *J* = 5.1, 12.5 Hz, 1H, H_e or $H_{e'}$), 2.81 (s, 4H, succinimida-H), 2.67 (t, *J* = 7.36 Hz, 2H, H_j), 2.58 (d, *J* = 12.4 Hz, 1H, $H_{e'}$ or H_e), 1.55 (m, 6H, H_h, H_i, H_j); ^{13}C NMR ([D6]dimethyl sulfoxide): δ = 173.3 (3C), 168.9, 162.7, 61.0, 59.2, 55.2, 30.0, 27.8, 27.6, 25.4 (2C), 24.3 ppm. These data is in concordance with *J. Med. Chem*. 52, **2009**, 7003-7013.

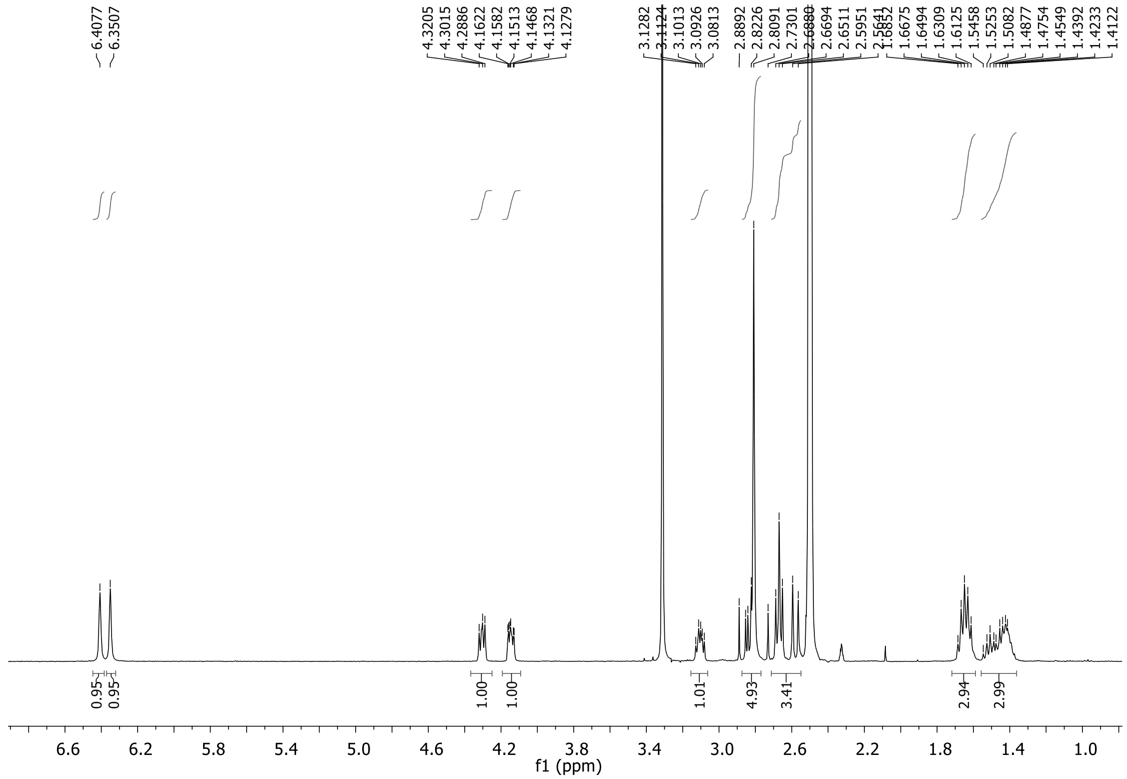
6.4077
6.3507
4.3205
4.3015
4.2886
4.1622
4.1582
4.1513
4.1468
4.1321
4.1279
3.1282
3.1124
3.1013
3.0926
3.0813
2.8892
2.8226
2.8091
2.7301
2.6880
2.6694
2.6511
2.5951
2.5641
1.6852
1.6675
1.6494
1.6309
1.6125
1.5458
1.5253
1.5082
1.4877
1.4754
1.4549
1.4392
1.4233
1.4122
0.95
0.95
1.00
1.00
1.01
4.93
3.41
2.94
2.99
6.6 6.2 5.8 5.4 5.0 4.6 4.2 3.8 3.4 3.0 2.6 2.2 1.8 1.4 1.0
f1 (ppm)

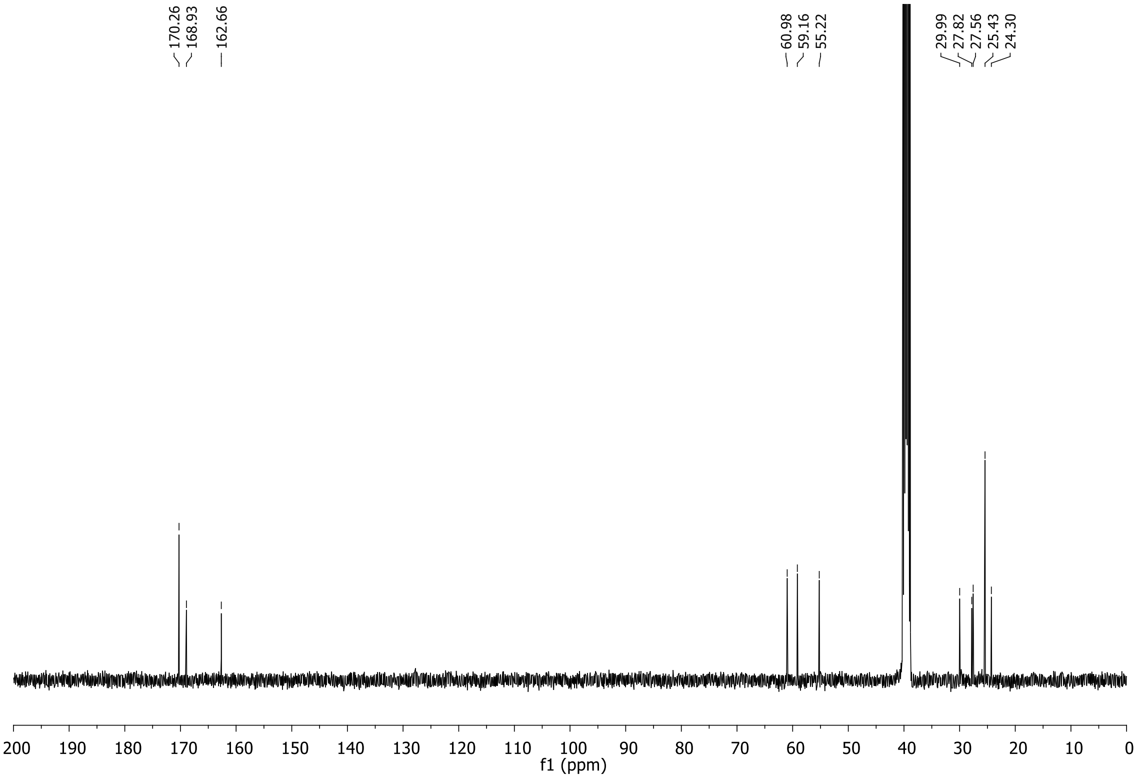
170.26
168.93
162.66
60.98
59.16
55.22
29.99
27.82
27.56
25.43
24.30
200 190 180 170 160 150 140 130 120 110 100 90 80 70 60 50 40 30 20 10 0
f1 (ppm)

2,2-diphenylethyl 6-([((9H-fluoren-9-yl)methoxy)carbonyl]amino)-2-[(*tert*-butoxy-carbonyl)amino)]hexanoate

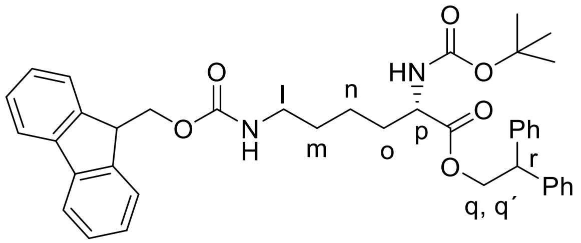

Compound 2

Boc-D-Lys(Fmoc)-OH (900 mg, 1.92 mmol) was dissolved in DCM (19 mL) and the solution was cooled to 0ºC, EDCI (732 mg, 3.84 mmol), 4-dimetylaminopyridine (DMAP) (catalytic amount) were added at 0ºC. The reaction mixture was allowed to stir at room temperature for 30 min, and then a solution of 2,2-diphenylethanol (457 mg, 2.3 mmol) in DCM (7.7 mL) was added to the activated acid. The reaction mixture was stirred overnight, concentrated under reduced pressure and then diluted with DCM. The organic layer was washed with 1M HCl, with $NaHCO_3$ (sat. aq.), then further washed with brine (sat. aq.), dried over Na_2SO_4 and concentrated under reduced pressure. The crude material was purified by column chromatography (eluent: hexane/AcOEt 3:1) to furnish the desired product as a colorless oil, 1.12g (quantitative yield). ^{1}H NMR ($CDCl_3$): δ = 7.77 (d, *J* = 7.5 Hz, 2H, Fmoc-H), 7.60 (d, *J* = 7.4 Hz, 2H, Fmoc-H), 7.40 (t, *J* = 8 Hz, 2H, Fmoc-H), 7.30 (m, 6 H, Ar-H), 7.22 (m, 6 H, Ar-H, Fmoc-H), 4.97 (m, 1 H, Fmoc-H), 4.80 (m, 1 H, H_q or $H_{q'}$), 4.68 (m, 1 H, NH), 4.58 (m, 1 H, H_q or $H_{q'}$), 4.41 (m, 3 H, H_r, CH_2-Fmoc), 4.22 (m, 2 H, H_p, NH), 3.05 (m, 2 H, H_l), 1.50 (m, 2H, H_m), 1.42 (s, 9 H, Boc-H), 1.07 (m, 2H, H_n); ^{13}C NMR ($CDCl_3$): δ= 172.5, 156.4, 155.3, 144.0 (2C), 141.3 (2C), 140.8, 140.6, 128.6 (2C), 128.6 (2C), 128.2 (2C), 128.1 (2C), 127.7 (2C), 127.0 (2C), 126.9 (2C), 125.0 (2C), 120.0 (2C), 79.9, 67.3, 66.4, 53.1, 49.8, 47.3, 40.6, 32.2, 29.7, 28.3, 22.1 (3C) ppm. MS m/z: calculated for $C_{40}H_{44}N_2O_6$ $[M+Na]^+$ 671.4 found MALDI-TOF 671.3.

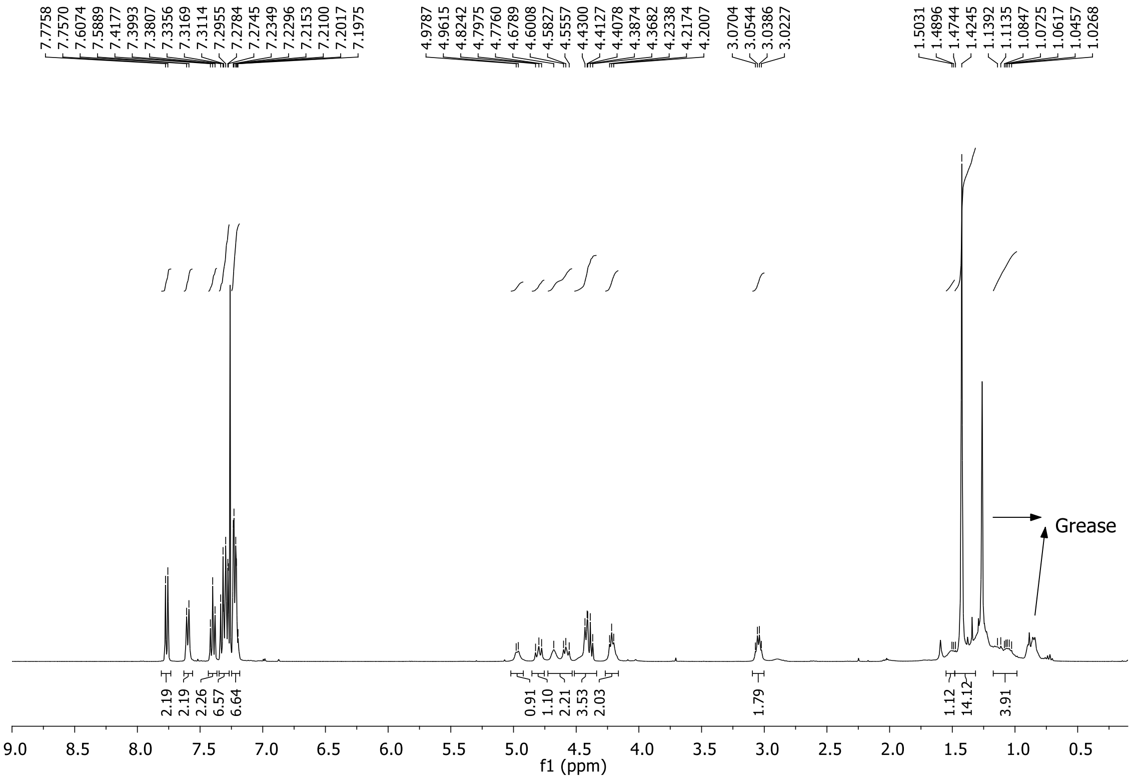
7.7758 7.7570 7.6074 7.5889 7.4177 7.3993 7.3807 7.3356 7.3169 7.3114 7.2955 7.2784 7.2745 7.2349 7.2296 7.2153 7.2100 7.2017 7.1975
4.9787 4.9615 4.8242 4.7975 4.7760 4.6789 4.6008 4.5827 4.5557 4.4300 4.4127 4.4078 4.3874 4.3682 4.2338 4.2174 4.2007
3.0704 3.0544 3.0386 3.0227
1.5031 1.4896 1.4744 1.4245 1.1392 1.1135 1.0847 1.0725 1.0617 1.0457 1.0268
Grease
2.19 2.19 2.26 6.57 6.64
0.91 1.10 2.21 3.53 2.03
1.79
1.12 14.12
3.91
9.0 8.5 8.0 7.5 7.0 6.5 6.0 5.5 5.0 4.5 4.0 3.5 3.0 2.5 2.0 1.5 1.0 0.5
f1 (ppm)

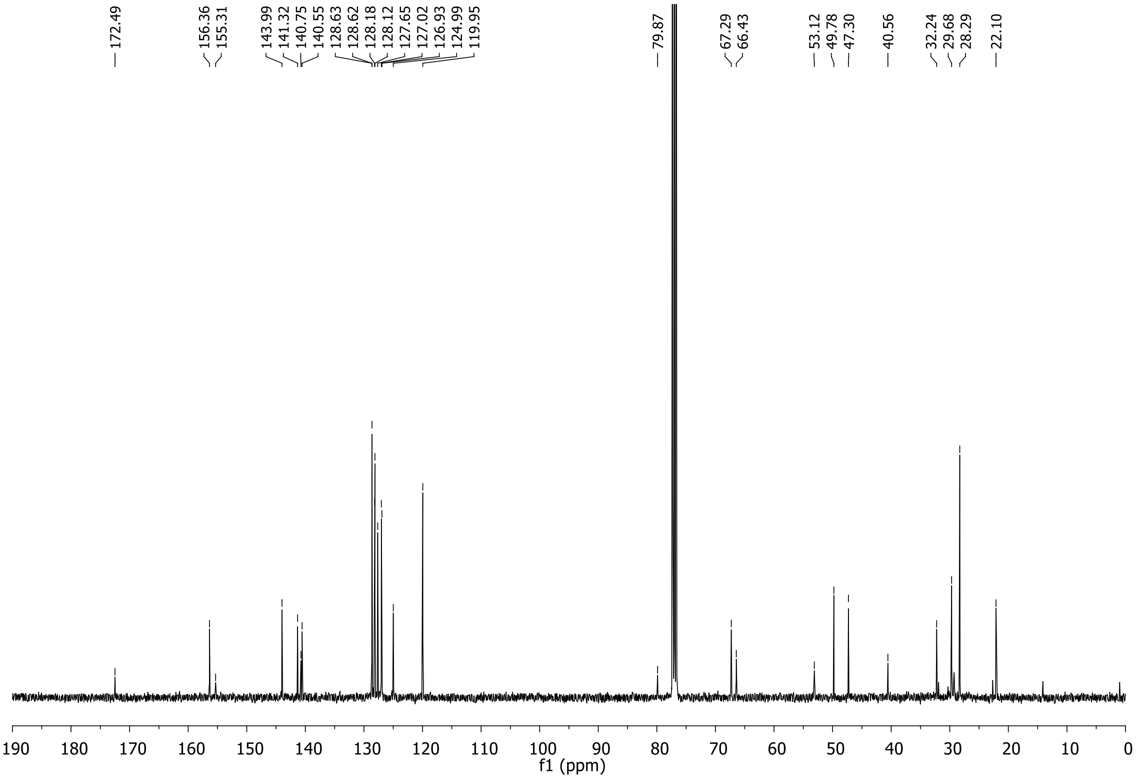
172.49
156.36 155.31
143.99 141.32 140.75 140.55 128.63 128.62 128.18 128.12 127.65 127.02 126.93 124.99 119.95
79.87
67.29 66.43
53.12 49.78 47.30
40.56
32.24 29.68 28.29
22.10
190 180 170 160 150 140 130 120 110 100 90 80 70 60 50 40 30 20 10 0
f1 (ppm)

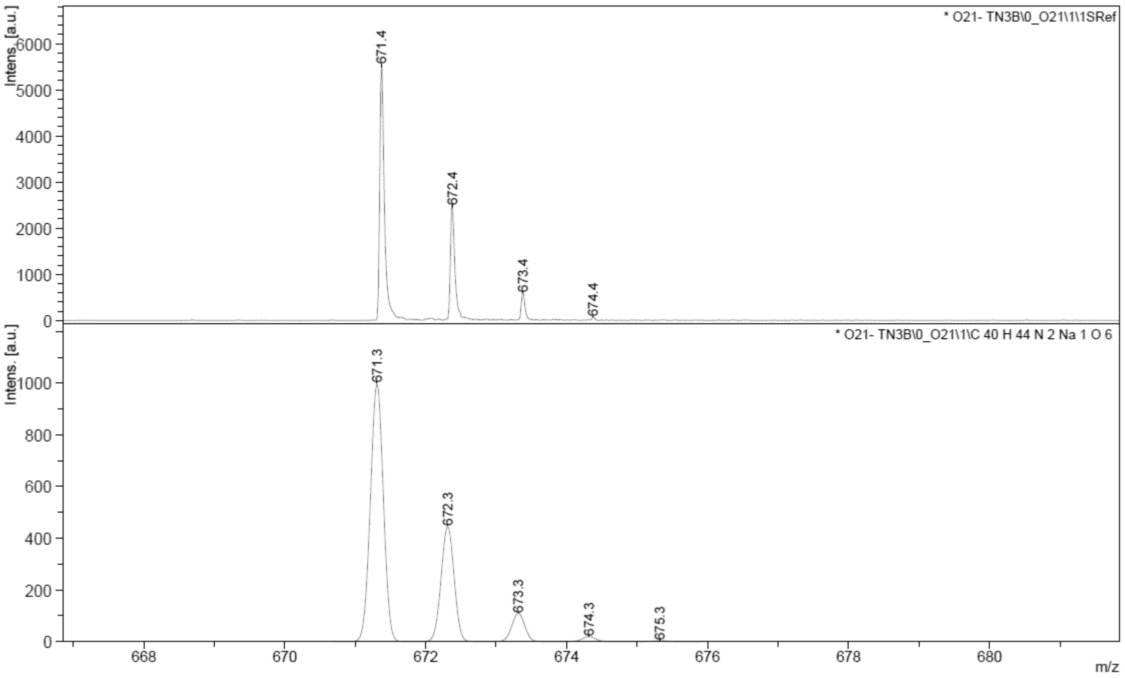

2,2-diphenylethyl 6-([((9H-fluoren-9-yl)methoxy)carbonyl]amino)-2-aminohexanoate

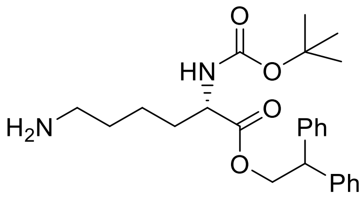

Compound 3

90mg (0.14 mmol) of compound **2** was dissolved in 1% piperidine and 1% DBU in DMF (2.8 mL). The reaction mixture was stirred for three hours at room temperature until the reaction was completed (TLC). The solvent was removed under reduced pressure to give compound **3** as yellowish oil (quantitative yield) and the crude material was used directly in the next step reaction.

2,2-diphenylethyl 2-[(tert-butoxycarbonyl)amino)-6-(biotinylamino] hexanoate

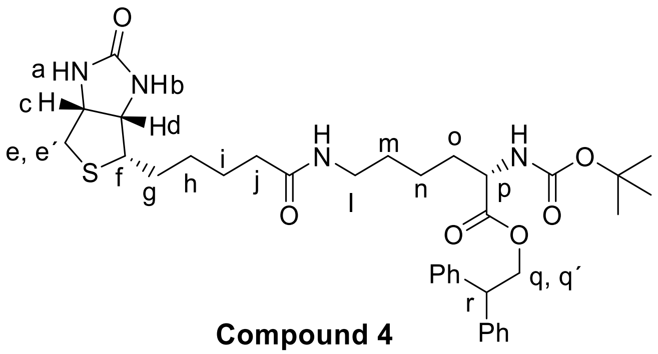

Compound 4

Compounds **3** (46 mg, 0.110 mmol) and **1** (25 mg, 0.073 mmol) were dissolved in DMF, and then Et_3N (20 μL, 0.144 mmol) was added. The reaction mixture was stirred overnight; then was concentrated in vacuum; the resulting residue was diluted in DCM and washed with water. The crude material was then purified by flash chromatography (using a gradient elution, from CH_3Cl to CH_3Cl/MeOH 9:1) to furnish **4** as a colorless oil, 45 mg, 96 %. ^1H NMR ($CDCl_3$): δ = 7.23 (m, 4 H, Ar-H), 7.14 (m, 6 H, Ar-H), 6.47 (br s, 1 H, H_a), 6.28 (br s, 1 H, H_b), 5.04 (br s, 1 H, NH), 4.71 (m, 1 H, , H_q or $H_{q'}$), 4.52 (dd, *J* = 7.3, 11.0 Hz, 1 H, $H_{q'}$ or H_q), 4.45 (dd, *J* = 4.8, 7.1 Hz, 1 H, H_c), 4.32 (t, *J* = 7.7 Hz, 1 H, H_r), 4.25 (dd, *J* = 4.5, 7.2 Hz, 1 H, H_d), 4.09 (br s, 1 H, H_p), 3.07 (m, 3 H, H_f, H_l), 2.83 (dd, *J* = 4.7, 12.9 Hz, 1 H, H_e or $H_{e'}$), 2.69 (d, *J* = 12.8 Hz, 1 H, $H_{e'}$ or $H_{e'}$), 2.17 (t, *J* = 6.8 Hz, 2 H, H_j), 1.61 (m, 4 H, H_g, H_i), 1.34 (s, 9 H, Boc-H), 1.11 (m, 8 H, H_h, H_n, H_m, H_o); ^{13}C NMR ($CDCl_3$): δ= 173.4, 172.6, 163.9, 155.5, 140.8, 140.6, 128.6 (2C), 128.6 (2C), 128.2 (2C), 128.1 (2C), 126.9 (2C), 79.9, 67.3, 61.9, 60.3, 55.6, 53.2, 49.8, 40.4, 39.1, 35.8, 32.2, 28.8, 28.3 (3C), 28.1, 28.0, 25.7, 22.4 ppm. MS m/z: calculated for $C_{35}H_{48}N_4O_6S$ $[M+Na]^+$ 675.3 found MALDI-TOF 675.4.

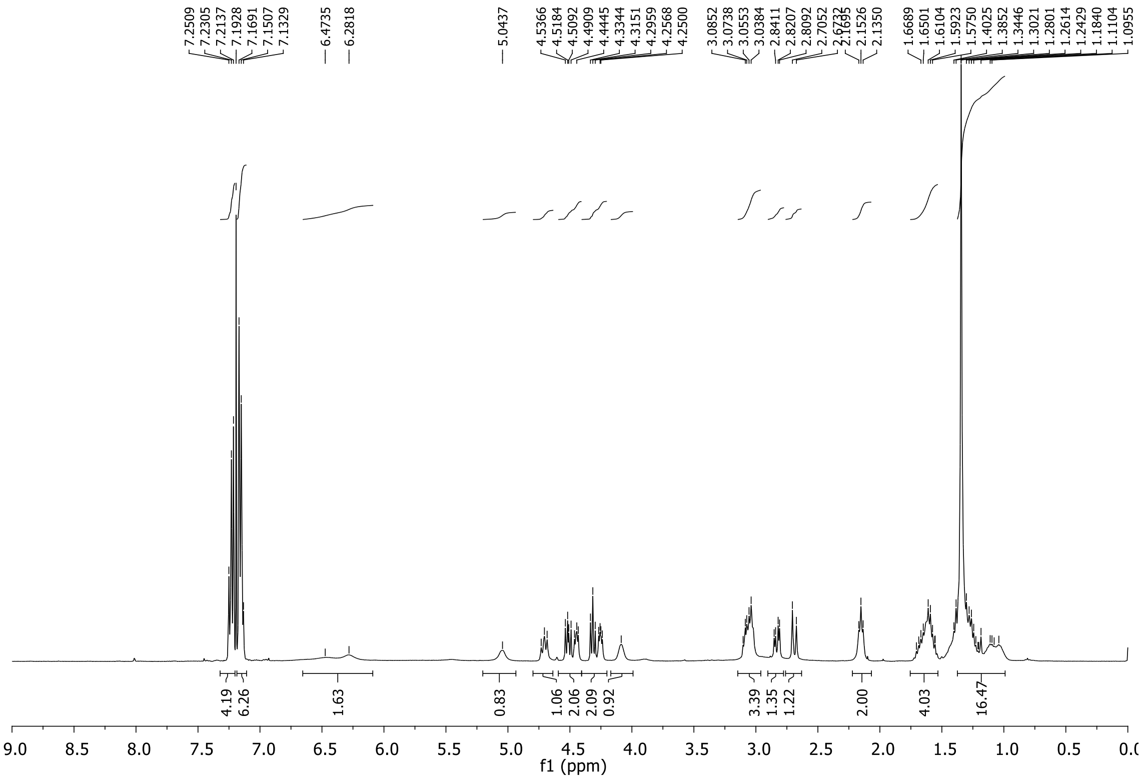

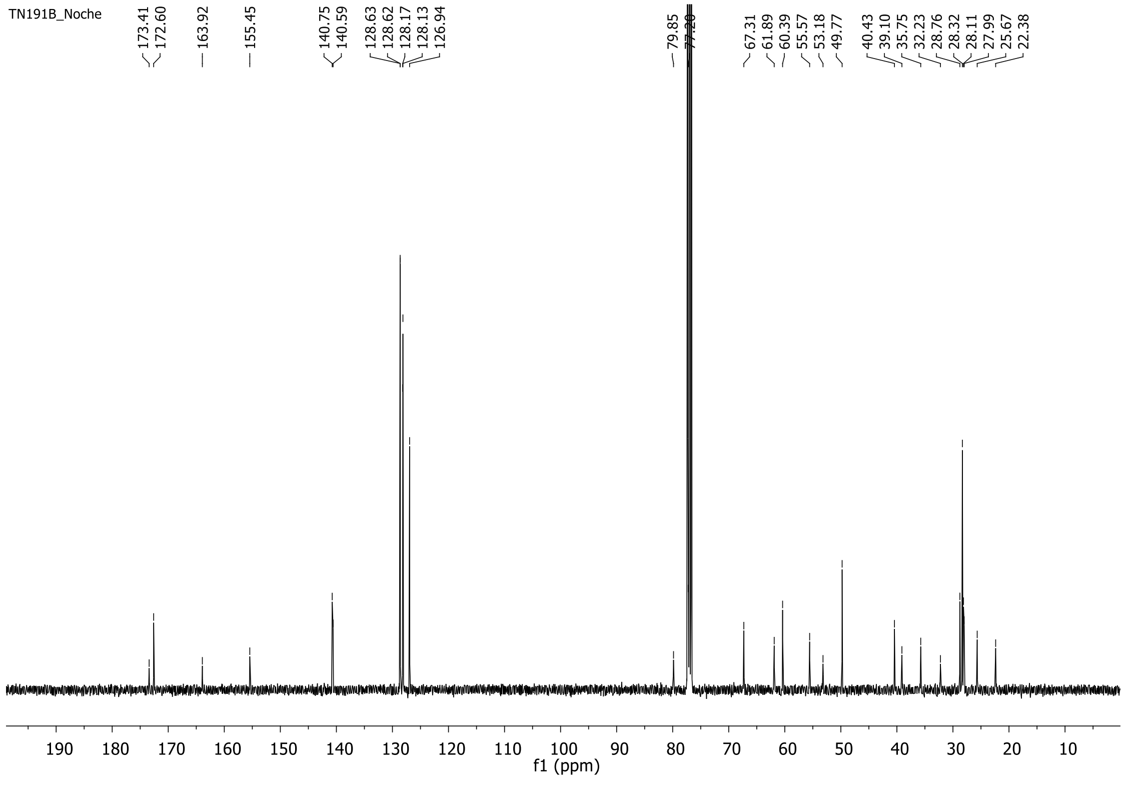

HSQC ($CDCl_3$)

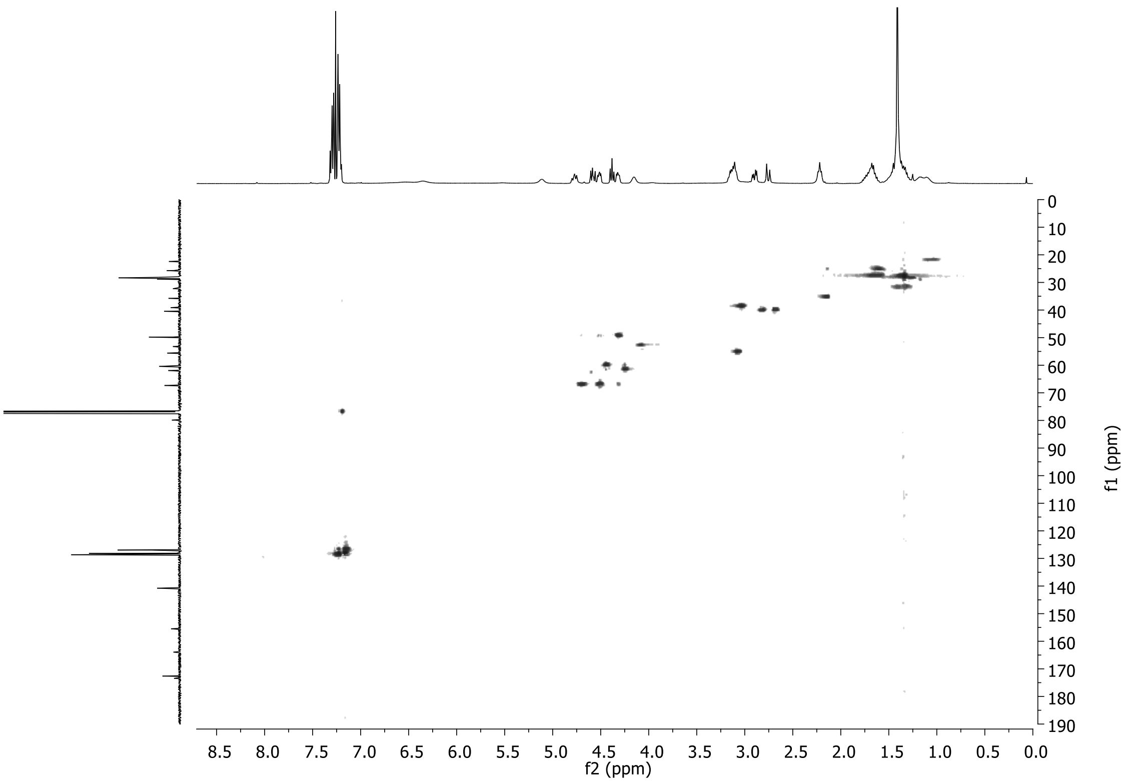

f2 (ppm)
f1 (ppm)

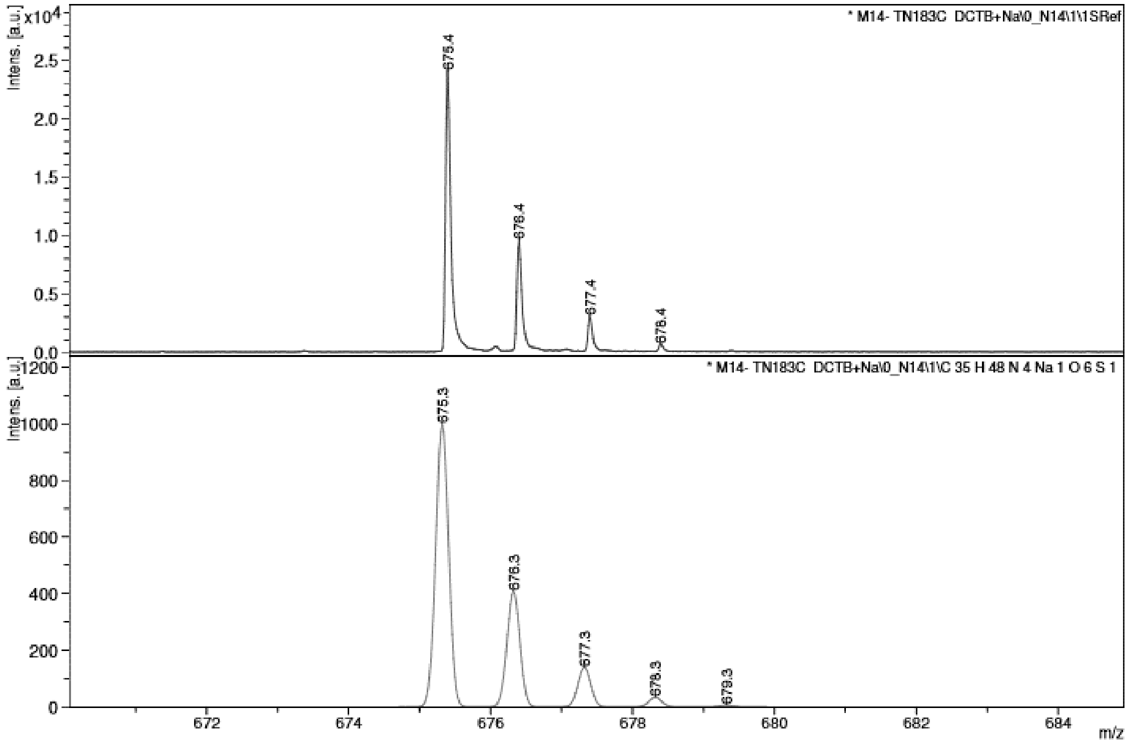

* M14- TN183C DCTB+Na\0_N14\1\1SRef
* M14- TN183C DCTB+Na\0_N14\1\C 35 H 48 N 4 Na 1 O 6 S 1
Intens. [a.u.]
m/z
675.4
676.4
677.4
678.4
675.3
676.3
677.3
678.3
679.3

1-(2,2-diphenylethoxy)-1-oxo-6-(biotinylamido)hexan-2-aminium 2,2,2-trifluoroacetate

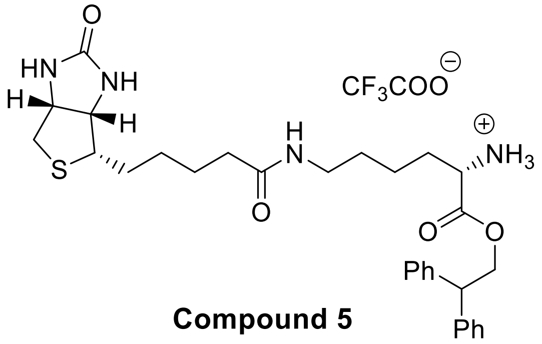

Compound 5

To a solution of compound **4** (58 mg, 0.09 mmol) in DCM (1.5 mL) TFA was added (0.1 mL) at 0ºC. The reaction was stirred at room temperature for 3h until it was completed (followed by TLC). The reaction mixture was concentrated under reduced pressure, then DCM was added and the organic layer was washed with aqueous $NaHCO_3$, further washed with brine, dried over Na_2SO_4 and concentrated under reduced pressure to give compound 5 as yellowish oil in quantitative yield. The crude material was used directly in the next reaction.

4-[(1-(2,2-diphenylethoxy)-6-(biotinylamino)-1-oxohexan-2-yl)amino]-4-oxobut-2-enoic acid

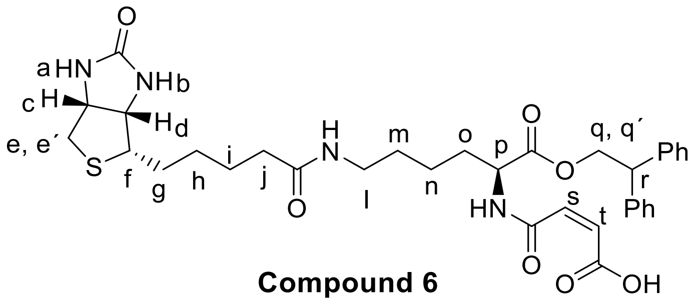

Compound 6

A solution of compound **5** (49 mg, 0.09 mmol) and Et_3N (12 μL, 0.093 mmol) in anhydrous THF (1 mL), was cooled to 0ºC, then maleic anhydride (9 mg, 0.093 mmol) dissolved in THF (0.4 mL) was added dropwise under nitrogen atmosphere. The solution was allowed to warm up to room temperature and stirred overnight. The solvent was then removed under reduced pressure and the crude material was purified by flash chromatography (using a gradient elution, starting DCM/MeOH 9:1 and then DCM/MeOH/NH_3 4:1:0.01), to furnish a yellow oil (59 mg 90% yield). ^1H NMR ([D6]dimethyl sulfoxide): δ = 11.09 (br s, 1H, OH), 7.91 (m, 1H, NH), 7.30 (m, 8 H, , Ar-H), 7.21 (m, 2 H, , Ar-H), 6.45 (br s, 1 H, H_a), 6.35 (br s, 1 H, H_b), 6.19 (d, J = 13.0 Hz, 1H, H_t), 5.84 (d, J = 12.8 Hz, 1H, H_s), 4.73 (dd, *J* = 7.9, 10.8 Hz, 1 H, H_q or $H_{q'}$), 4.53 (dd, *J* =

7.2, 10.9 Hz, 1 H, $H_{q'}$ or H_q), 4.36 (t, J = 7.6 Hz, 1H, H_r), 4.29 (m, 1 H, H_c), 4.10 (m, 2 H, H_d, H_p), 3.08 (m, 1 H, H_f), 2.92 (m, 2 H, H_l), 2.81 (dd, *J* = 4.7, 12.9 Hz, 1 H, H_e or $H_{e'}$), 2.56 (d, *J* = 12.8 Hz, 1 H, $H_{e'}$ or H_e), 2.07 (t, *J* = 6.8 Hz, 2 H, H_j), 1.44 (m, 12 H, H_g, H_i, H_h, H_n, H_m, H_o); ^{13}C NMR ([D6]dimethyl sulfoxide): δ = 172.0, 171.7, 167.2, 165.3, 162.7, 141.3, 141.2, 136.2, 128.5 (2C), 128.5 (2C), 128.2, 128.0 (4C), 126.7 (2C), 66.3, 61.1, 59.2, 55.4, 52.3, 49.3, 39.9, 37.7, 35.2, 30.2, 28.6, 28.2, 28.0, 25.4, 22.2 ppm. MS m/z: calculated for $C_{34}H_{42}N_4O_7S$ $[M+H]^+$ 650.8 found FAB 651.3.

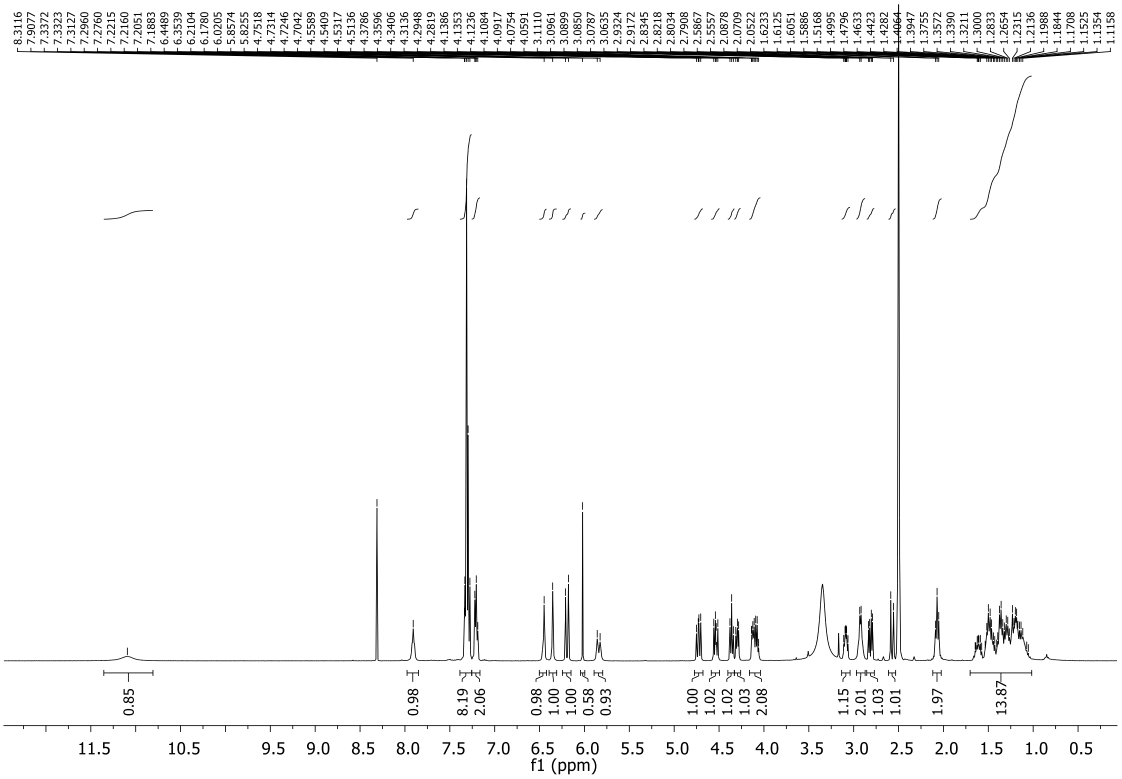

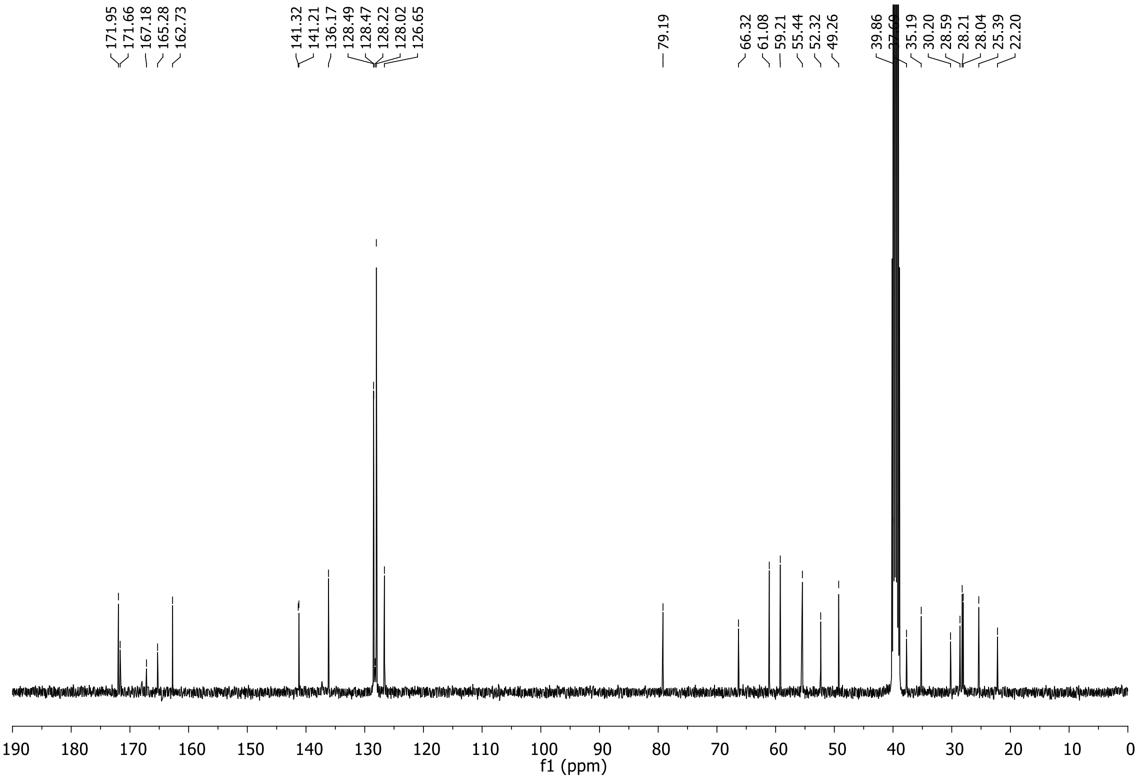

COSY ([D6]dimethyl sulfoxide)

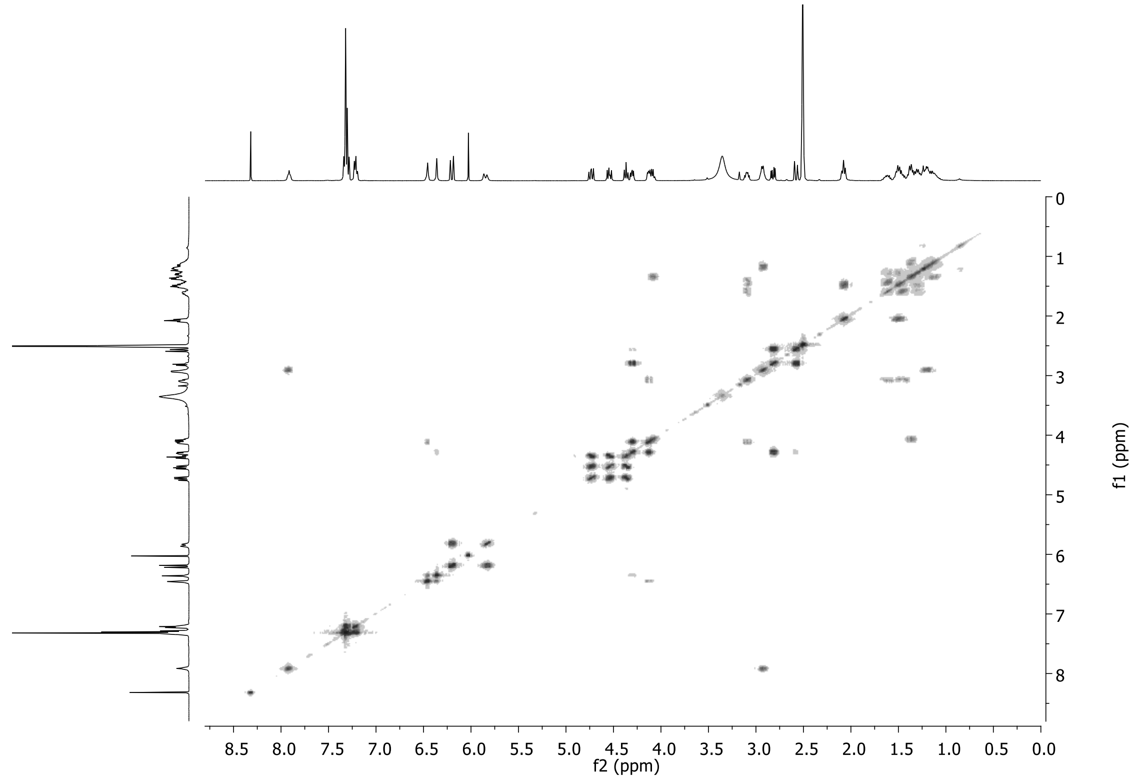

HSQC ([D6]dimethyl sulfoxide)

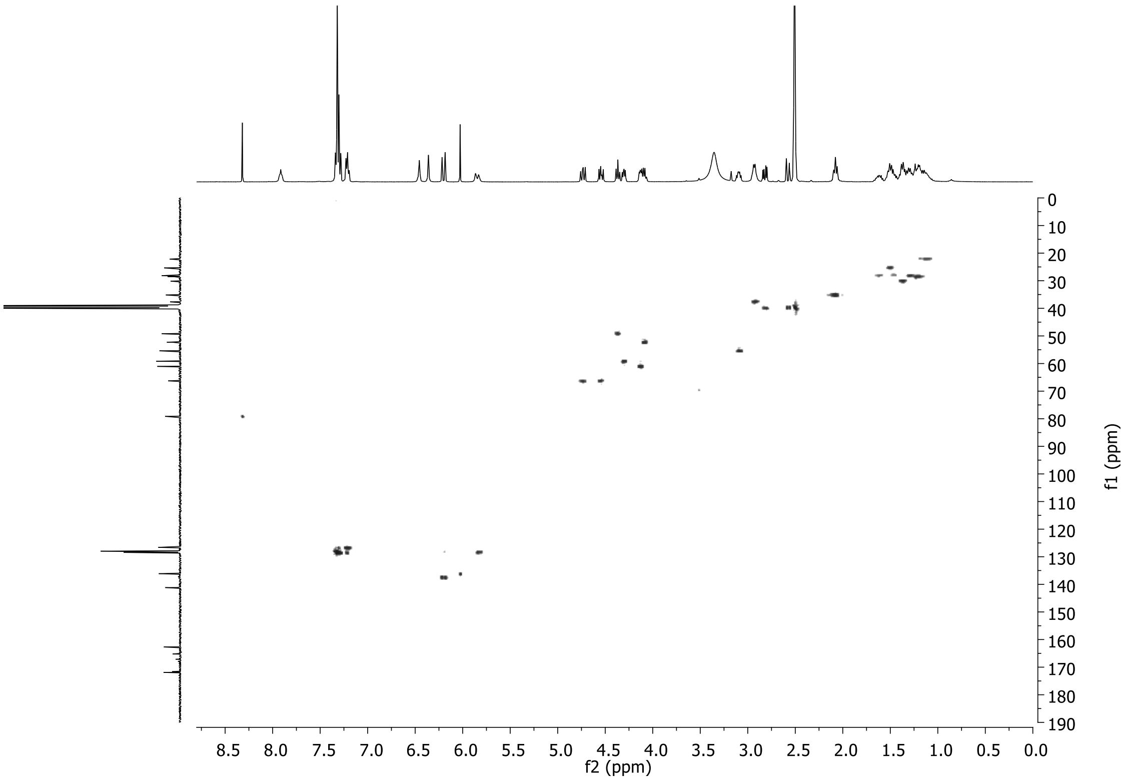

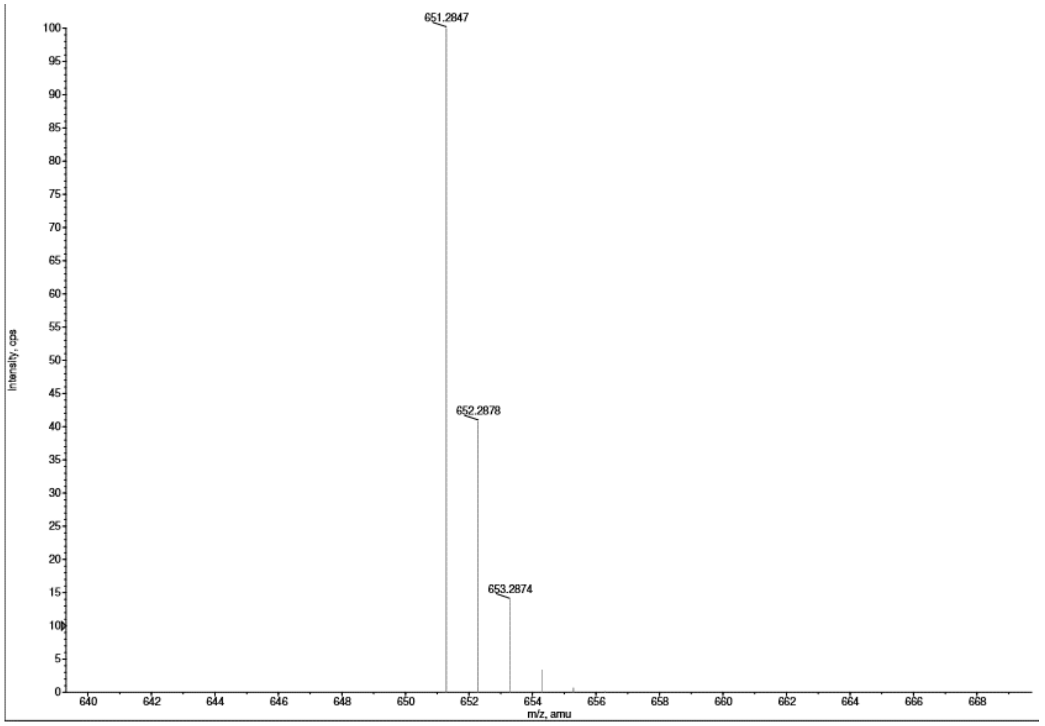

2,2-Diphenylethyl succinic acid mono ester

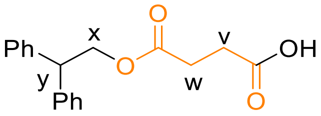

Compound 7

To a stirred solution of 2,2,-diphenylethanol (1 g, 5.26 mmol) in DCM (53mL) was added one drop of Et_3N and a solution of succinic anhydride (584 mg, 5.83 mmol) in 15 mL DCM, added slowly over 30 min. The reaction mixture was stirred overnight. Once the reaction was completed (follow by TLC), the solvent was removed under reduced pressure and the crude material was purified by column chromatography, eluent: Hexane/AcOEt 2:1, to afford 1.2 g of compound **7** as a white solid (76%). ^{1}H NMR ($CDCl_3$): δ = 7.28 (m, 4H, , Ar-H), 7.21 (m, 6H, Ar-H), 4.65 (d, J = 7.6 Hz, 2H, H_x), 4.36 (t, J = 7.6 Hz, 1H, H_y), 2.55 (m, 4 H, H_v, H_w) ppm.

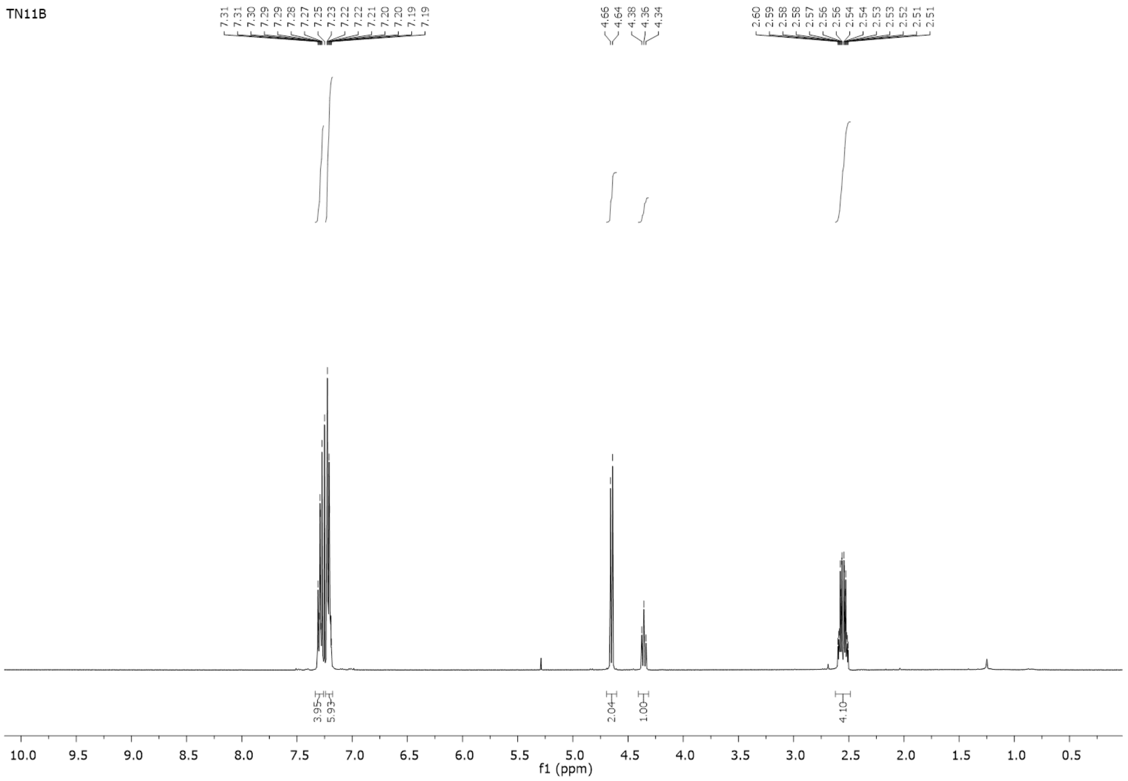

tert-Butyl (2-(2-(2-aminoethyl) hexacosaethylenoxy)ethyl carbamate

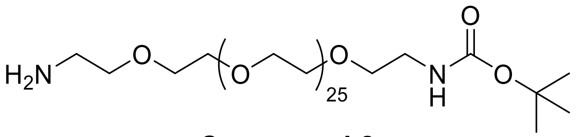

Compound 8

The reaction was carried out under anhydrous conditions. To 490mg (0.4 mmol) *O,O´*-bis (2-aminoethyl) hexacosaethylene glycol (EG26) in DCM at 0ºC was added di-tert-butyl dicarbonate (44mg, 0.2 mmol). The solution was stirred overnight, then the solvent was removed in vacuo and the crude material purified via column chromatography using a gradient elution, starting with $CHCl_3$/MeOH 10:1 to 4:1 and finally 1:1, to give a yellowish oil, 165 mg, 61%. ^{1}H NMR ($CDCl_3$): δ = 7.91 (br s 3H, 3NH), 3.92 (m, 2H), 3.63 (m, 106H, EG26-H), 3.52 (t, *J* = 5.1, 2H), 3.29 (t, *J* = 5.2, 2H), 3.17 (m, 2H), 1.43 (s, 9H, Boc-H); ^{13}C NMR ($CDCl_3$): δ = 154.9, 79.0, 70.5 (51C), 70.3, 70.0, 69.8, 40.4, 29.7, 40.4 (3C) ppm. MS m/z: calculated for $C_{61}H_{124}N_2O_{29}$ $[M+Na]^+$ 1371.8 found MALDI-TOF 1371.8.

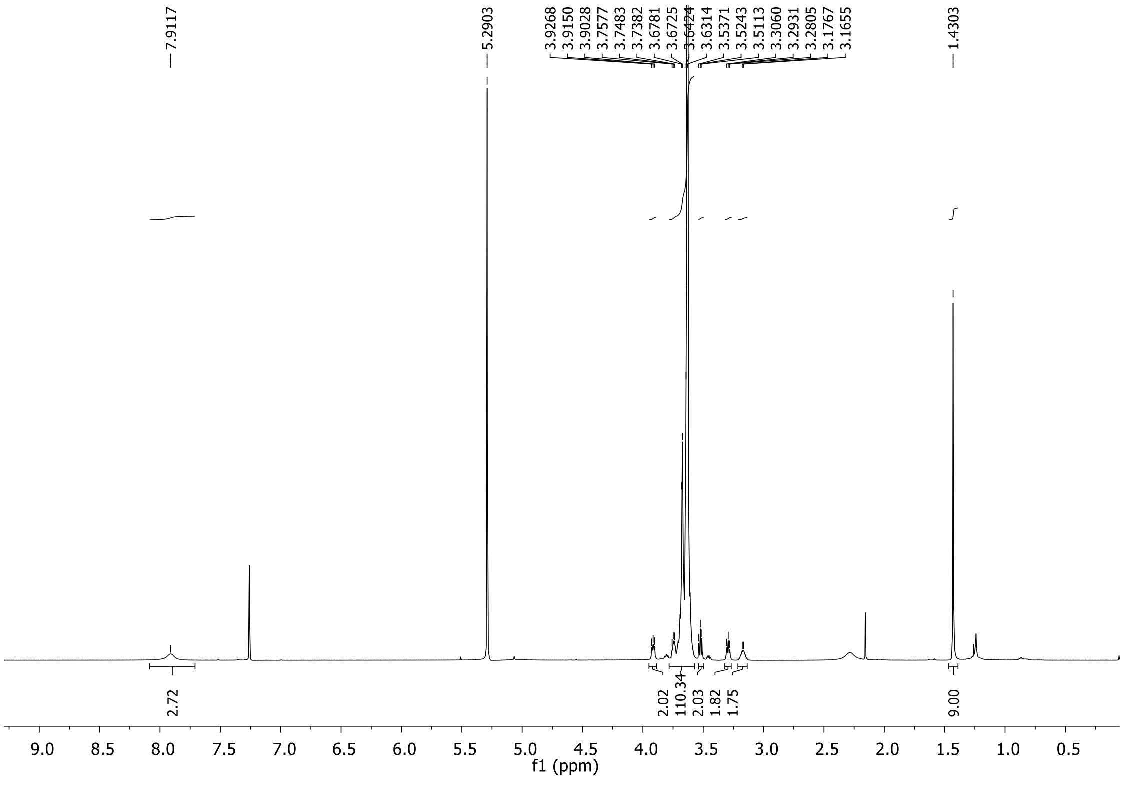

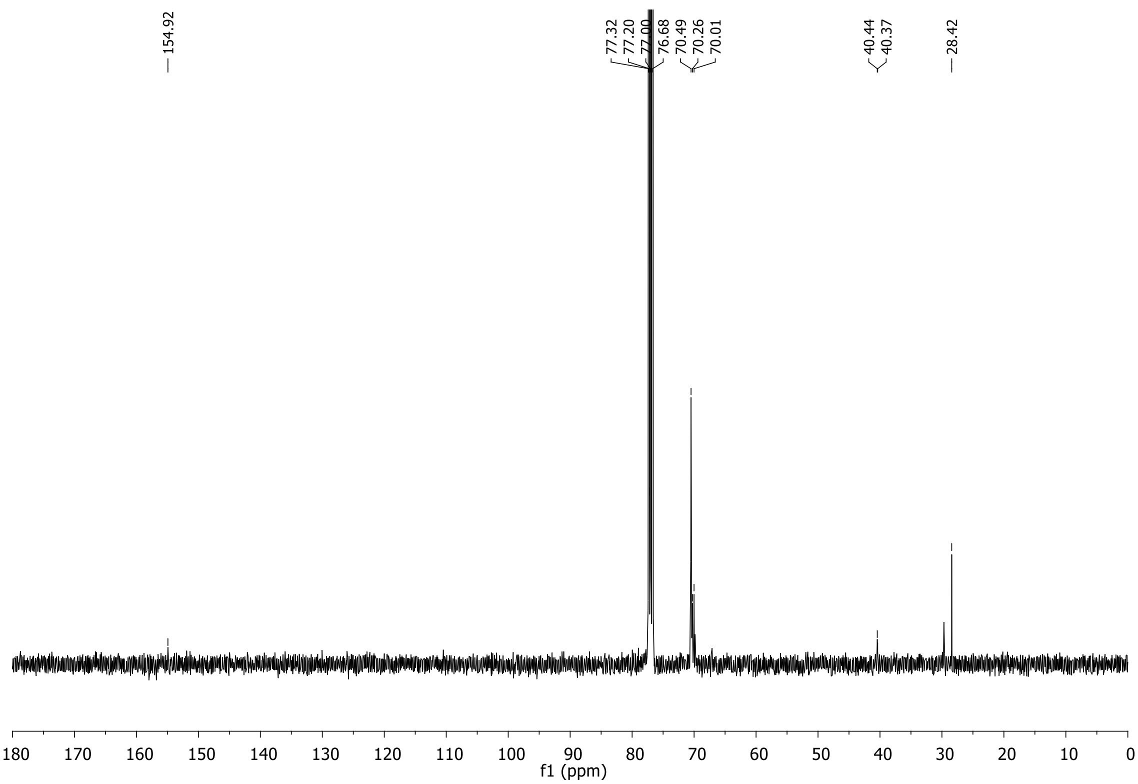
154.92
77.32
77.20
77.00
76.68
70.49
70.26
70.01
40.44
40.37
28.42
180 170 160 150 140 130 120 110 100 90 80 70 60 50 40 30 20 10 0
f1 (ppm)

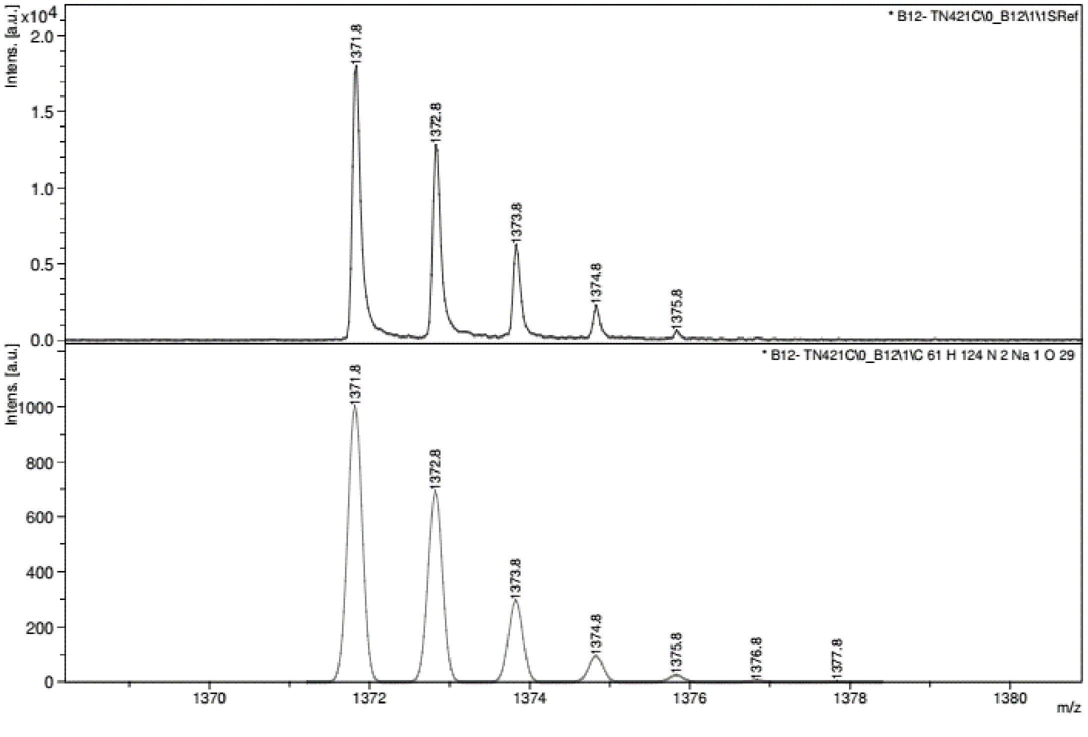
* B12- TN421C\0_B12\1\1SRef
Intens. [a.u.]
x10^4
2.0
1.5
1.0
0.5
0.0
1371.8
1372.8
1373.8
1374.8
1375.8
* B12- TN421C\0_B12\1\C 61 H 124 N 2 Na 1 O 29
1000
800
600
400
200
0
1376.8
1377.8
1370 1372 1374 1376 1378 1380
m/z

2,2-diphenylethyl 2,2-dimethyl-4,18-dioxo-3,8,11,14-tetraoxa-5,17-diazahenicosan-21-oate

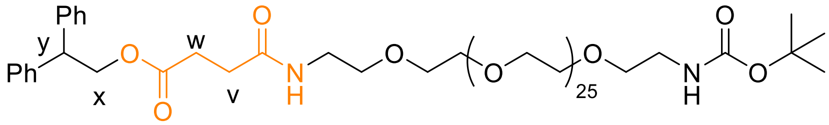

Compound 9

40 mg (0.13 mmol) of compound **7** was dissolved in DCM (2 mL) and the solution was cooled down to 0ºC. Then EDCI (38 mg, 0.2 mmol) and DMAP (24 mg, 0.2 mmol) were added at 0ºC and the reaction mixture was allowed to stir at room temperature for 30 min. A solution of compound **8** (165 mg, 0.12 mmol), in DCM (1.5 mL) was added to the activated acid. The reaction mixture was stirred for 24h, concentrated under reduced pressure and then diluted with DCM and washed with 1M HCl, with $NaHCO_3$ (sat. aq.). The organic layer was further washed with brine (sat.), dried over Na_2SO_4 and concentrated under reduced pressure. The crude material was purified by column chromatography using $CHCl_3$/MeOH 15:1 as eluent, to give compound **9** as a colorless oil, 180mg, 92%. ^{1}H NMR ($CDCl_3$): δ = 7.28 (m, 4H, Ar-H), 7.21 (m, 6H, Ar-H), 6.23 (br s, 1H, NH), 5.03 (br s, 1H, NH), 4.62 (d, J = 7.6 Hz, 2H, H_x), 4.35 (t, J = 7.6 Hz, 1H, H_y), 3.81 (m, 1H), 3.63 (m, 103H, EG26-H), 3.53 (m, 4H), 3.40 (m, 2H), 3.30 (m, 2H), 2.57 (t, 2H, J = 7.2 Hz, H_w), 2.37 (t, 2H, J = 7.0 Hz, H_v),1.43 (s, 9H, Boc-H); ^{13}C NMR ($CDCl_3$): δ = 172.7, 171.5, 156.0, 141.1 (2C), 128.5 (4C), 128.2 (4C), 126.8 (2C), 79.2, 70.6 (51C), 70.2, 70.2, 69.7, 66.8, 49.8, 40.5, 39.4, 30.6, 29.7, 28.4 (3C) ppm. MS m/z: calculated for $C_{79}H_{140}N_2O_{32}$ $[M+Na]^+$ 1651.9 found MALDI-TOF 1651.9.

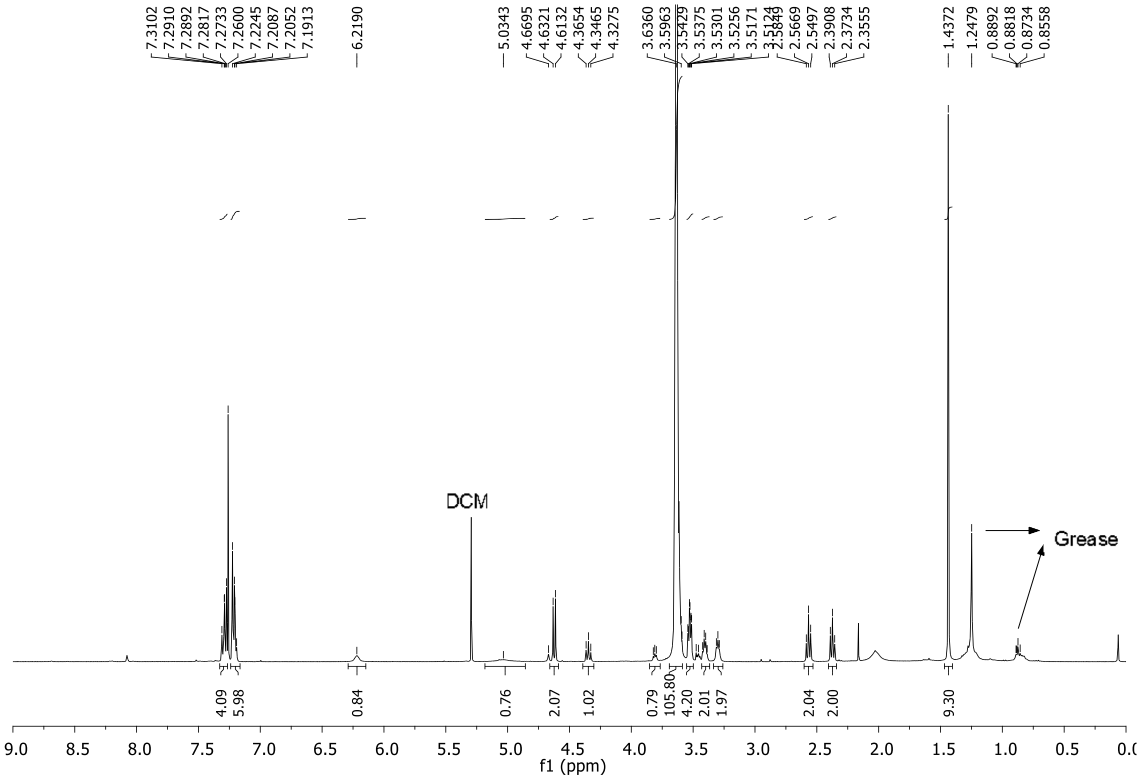
7.3102
7.2910
7.2892
7.2817
7.2733
7.2600
7.2245
7.2087
7.2052
7.1913
6.2190
5.0343
4.6695
4.6321
4.6132
4.3654
4.3465
4.3275
3.6360
3.5963
3.5429
3.5375
3.5301
3.5256
3.5171
2.5669
2.5497
2.3908
2.3734
2.3555
1.4372
1.2479
0.8892
0.8818
0.8734
0.8558
DCM
Grease
4.09
5.98
0.84
0.76
2.07
1.02
0.79
105.80
4.20
2.01
1.97
2.04
2.00
9.30
f1 (ppm)

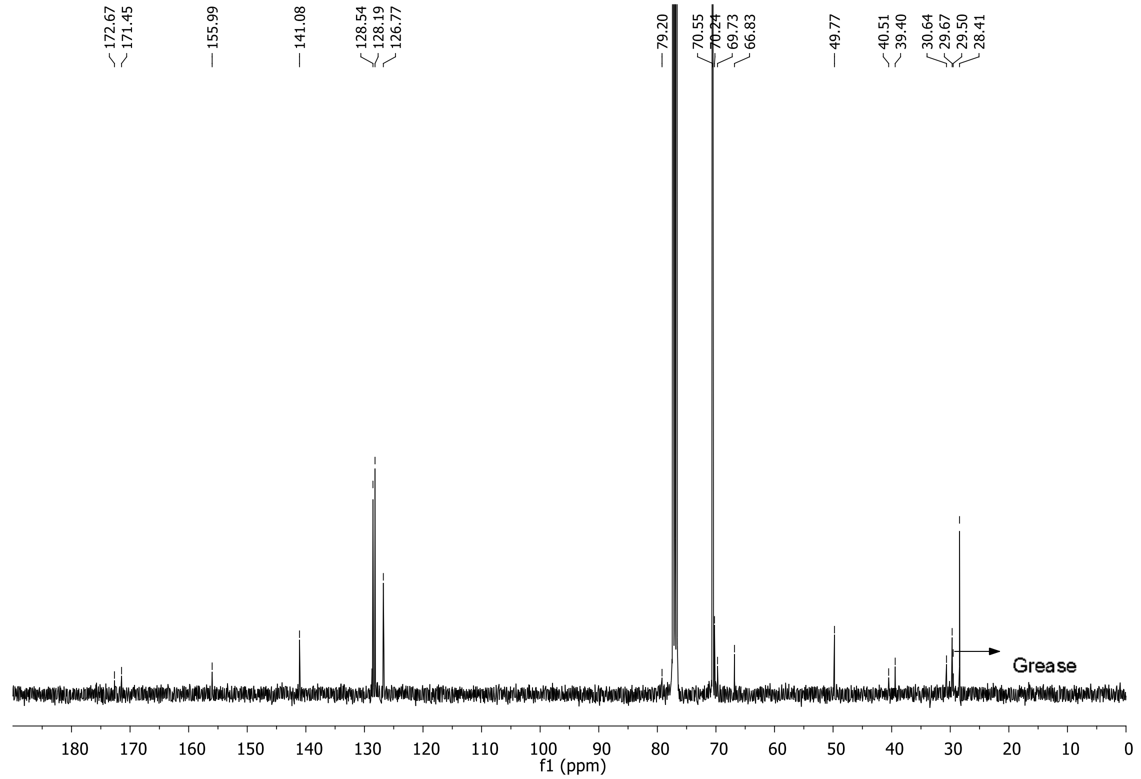
172.67
171.45
155.99
141.08
128.54
128.19
126.77
79.20
70.55
69.73
66.83
49.77
40.51
39.40
30.64
29.67
29.50
28.41
Grease
f1 (ppm)

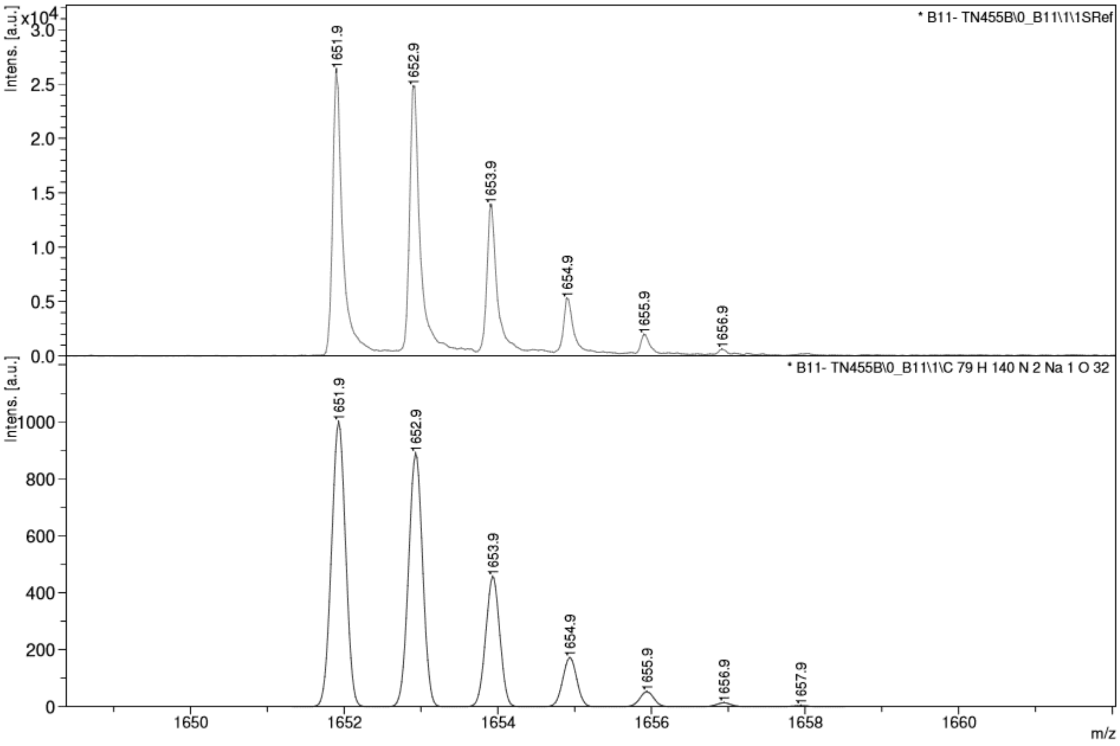

Compound 10 was synthetized following the same procedure described for compound 5.

(Z/E 55/45)-Thread

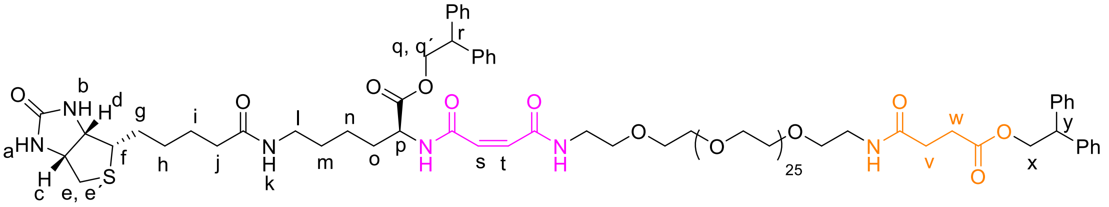

Compound 11

Compound **6** (85 mg, 0.13 mmol) was dissolved in DCM (3 mL) and the solution was cooled to 0ºC, EDCI (23 mg, 0.19 mmol), DMAP (37 mg, 0.19 mmol) were added at 0ºC. The reaction mixture was allowed to stir at room temperature for 30 min, and then a solution of compound **10** (180 mg, 0.12 mmol) in DCM (7.7 mL) was added to the activated acid. The reaction mixture was stirred overnight, concentrated under reduced pressure and then diluted with DCM. The organic layer was washed with 1M HCl, with $NaHCO_3$ (sat. aq.), then further washed with brine (sat. aq.), dried over Na_2SO_4 and concentrated under reduced pressure. The crude material was purified by column chromatography (gradient elution: DCM/MeOH 30:1 to 9:1) to furnish the

desired product as a colorless oil, 166mg, 0.077 mmol (64%).^{1}H NMR ([D6]dimethyl sulfoxide): δ = 8.65 (d, *J* = 7.2 Hz, 1H, NH), 8.50 (t, *J* = 5.6 Hz, 1H, NH), 7.86 (t, *J* = 5,5 Hz, 1H, NH), 7.70 (t, *J* = 5,5 Hz, 1H, NH), 7.27 (m, 16 H, Ar-H), 7.21 (m, 4 H, Ar-H), 6.87 (d, J = 15.5 Hz, 0.4 H, H_r or H_s of *E*-isomer), 6.82 (d, J = 15.5 Hz, 0.4 H, H_r or H_s of *E*-isomer), 6.40 (br s, 1 H, H_a), 6.34 (br s, 0.5 H, H_b), 6.21 (d, *J* = 13.1 Hz, 0.5 H, H_r or H_s of *Z*-isomer), 6.08 (d, *J* = 13.1 Hz, 1H, H_s or H_t of *Z*-isomer), 4.72 (dd, *J* = 8.1, 10.9 Hz, 1 H, H_q or $H_{q'}$), 4.55 (m, 3H, $H_{q'}$ or H_q, H_x), 4.33 (m, 3H, H_c, H_y, H_r), 4.12 (m, 2H, H_d, H_p), 3.68 (m, 1H, EG26-H), 3.50 (m, 107H, EG26-H), 3.37 (t, *J* = 6.0 Hz, 2H, EG26-H), 3.17 (dd, *J* = 5.8, 11.5 Hz, 2H, EG26-H), 3.08 (m, 1H, H_f), 2.91 (m, 2 H, H_l), 2.81 (dd, *J* = 4.7, 12.9 Hz, 1 H, H_e or $H_{e'}$), 2.54 (d, *J* = 12.8 Hz, 1 H, $H_{e'}$ or H_e), 2.37 (t, *J* = 6.9 Hz, 2 H, H_w), 2.27 (t, *J* = 6.6 Hz, 2 H, H_v), 2.04 (t, *J* = 6.8 Hz, 2 H, H_j), 1.44 (m, 12 H, H_g, H_i, H_h, H_n, H_m, H_o) (400 MHz, DMSO) ppm. MS m/z: calculated for $C_{108}H_{172}N_6O_{36}S$ $[M+Na]^+$ 2185.1 found MALDI-TOF 2185.1.

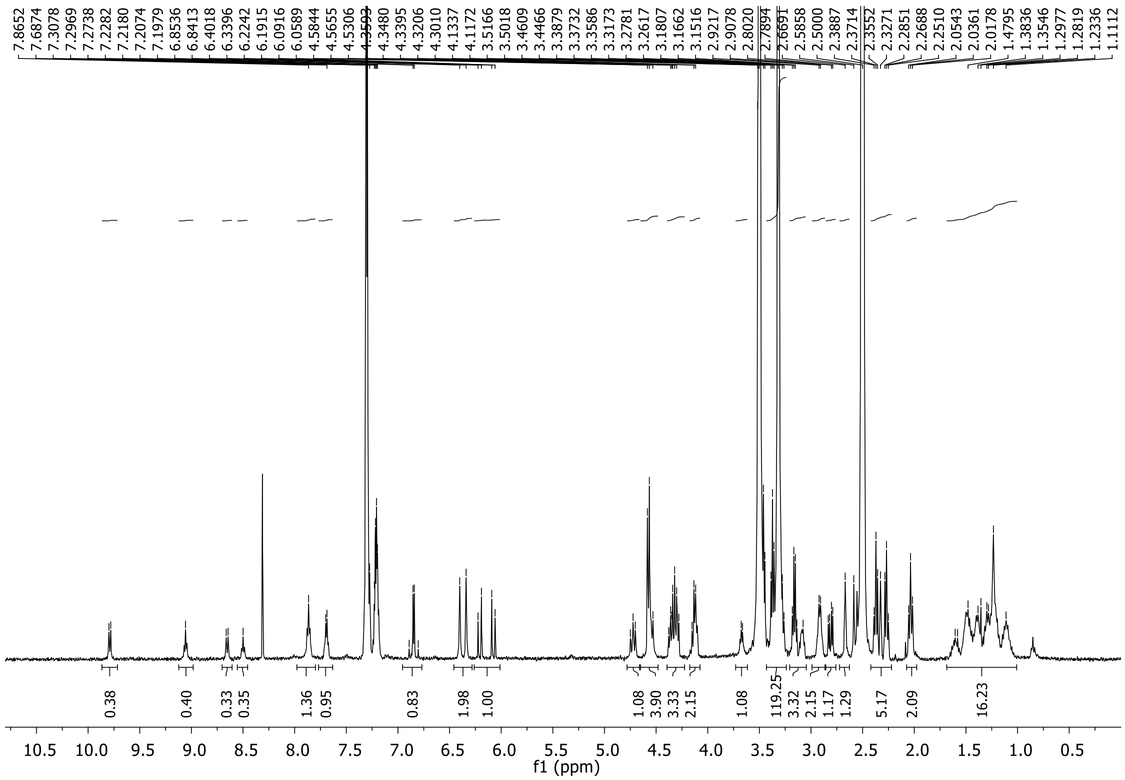

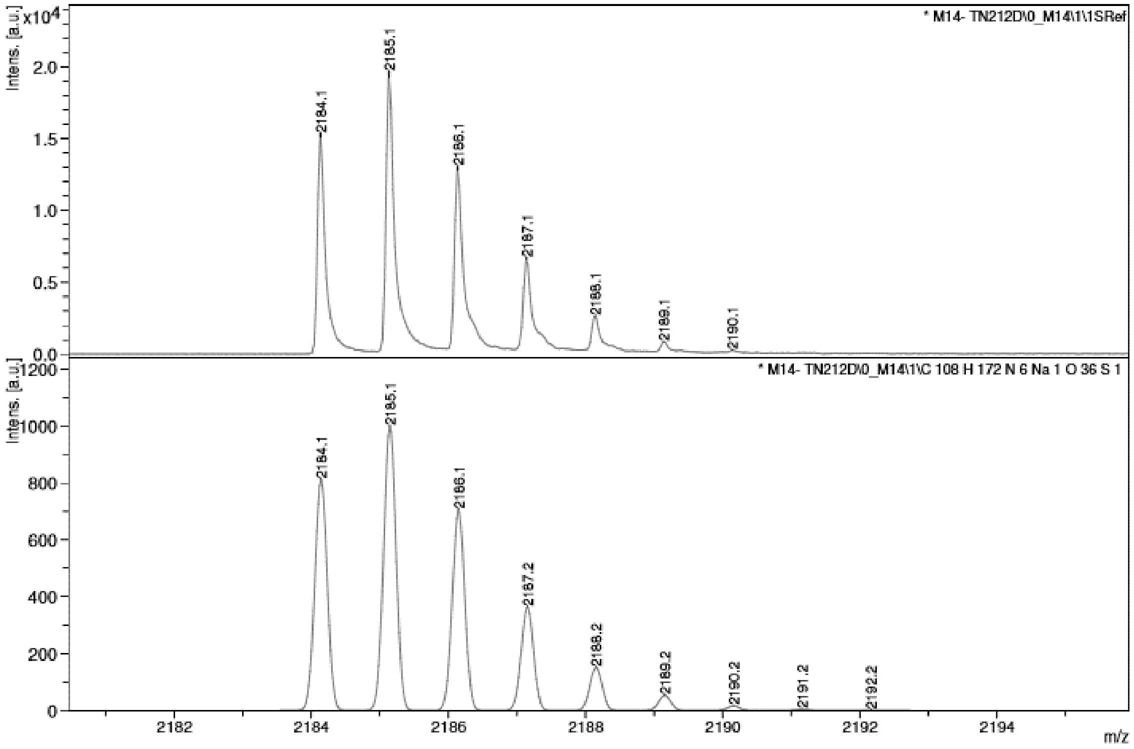

<u>*(E)*-Thread</u>

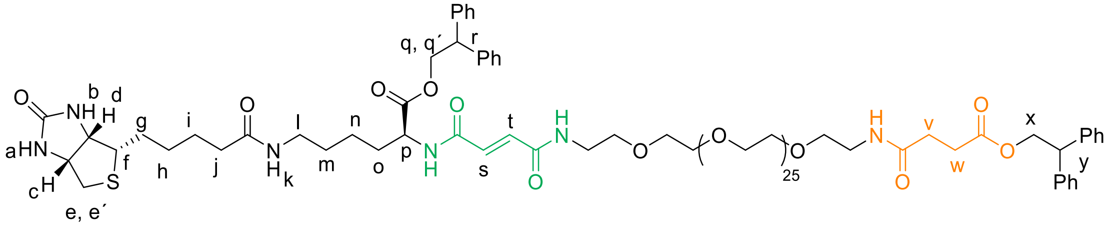

Compound 12

A solution of compound **11** (115 mg, 0.052mmol) and piperidine (4 μg, 0.006 mmol) in DCM (3 mL) was stirred for 48 h. Then DCM was added and the organic layer was washed twice with 1M HCl. The organic layer was concentrated under reduced pressure to furnish compound **12** as yellowish oil (112 mg, 97%). ^{1}H NMR ([D6]dimethyl sulfoxide): δ = 8.65 (d, *J* = 7.2 Hz, 1H, NH), 8.50 (t, *J* = 5.6 Hz, 1H, NH), 7.86 (t, *J* = 5,5 Hz, 1H, NH), 7.70 (t, *J* = 5,5 Hz, 1H, NH), 7.27 (m, 16 H, Ar-H), 7.21 (m, 4 H, Ar-H), 6.87 (d, J = 15.5 Hz, 1H, H_s or H_t), 6.82 (d, J = 15.5 Hz, 1H, H_s or H_t), 6.40 (br s, 1 H, H_a), 6.34 (br s, 1 H, H_b), 4.72 (dd, *J* = 8.1, 10.9 Hz, 1 H, H_p or $H_{p'}$), 4.55 (m, 3H, $H_{q'}$ or H_q, H_x), 4.33 (m, 3H, H_c, H_y, H_r), 4.12 (m, 2H, H_d, H_p), 3.68 (m, 1H, EG26-H), 3.50 (m, 107H, EG26-H), 3.37 (t, *J* = 6.0 Hz, 2H, EG26-H), 3.17 (dd, *J* = 5.8, 11.5 Hz, 2H, EG26-H), 3.08 (m, 1H, H_f), 2.91 (m, 2 H, H_l), 2.81 (dd, *J* = 4.7, 12.9 Hz, 1 H, H_e or $H_{e'}$), 2.54 (d, *J* = 12.8 Hz, 1 H, $H_{e'}$ or H_e), 2.37 (t, *J* = 6.9 Hz, 2 H, H_w), 2.27 (t, *J* = 6.6 Hz, 2 H, H_v),

2.04 (t, J = 6.8 Hz, 2 H, H_j), 1.44 (m, 12 H, H_g, H_i, H_h, H_n, H_m, H_o); ^{13}C NMR ([D6]dimethyl sulfoxide): δ = 173.4, 172.7, 171.9, 171.2, 165.4, 164.6, 163.6, 141.1 (2C), 140.8, 140.8, 140.6, 140.5, 133.4, 132.4, 129.7, 128.6 (2C), 128.6 (2C), 128.5 (4C), 128.3, 128.2 (4C), 128.1 (2C), 126.9 (2C), 126.7(2C), 70.5 (50C), 70.5, 70.2, 69.8, 69.6, 67.3, 66.7, 55.5, 52.3, 49.7, 49.7, 40.4, 39.6, 39.3, 38.3, 35.7, 30.9, 30.7, 29.6, 29.4, 28.7, 28.0, 25.5, 22.6, 21.9 ppm. MS m/z: calculated for $C_{108}H_{172}N_6O_{36}S$ $[M+Na]^+$ 2184.0 found FAB 2184.1.

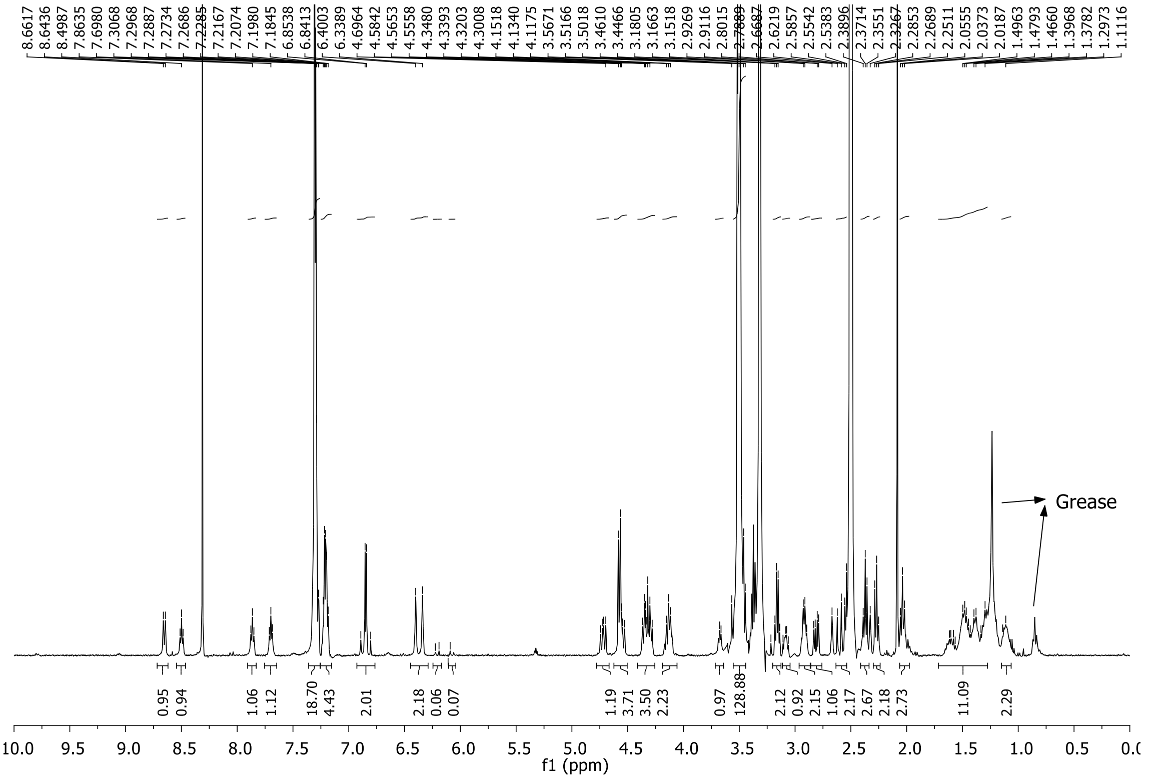

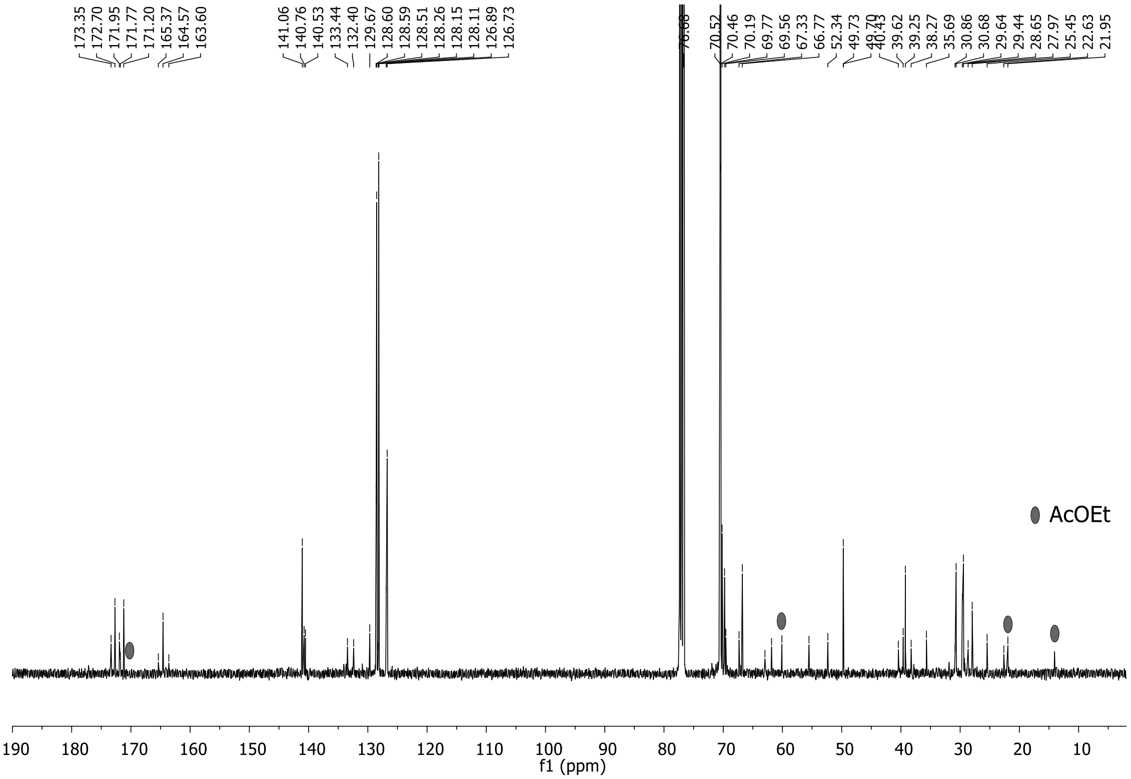
173.35
172.70
171.95
171.77
171.20
165.37
164.57
163.60
141.06
140.76
140.53
133.44
132.40
129.67
128.60
128.59
128.51
128.26
128.15
128.11
126.89
126.73
70.52
70.46
70.19
69.77
69.56
67.33
66.77
52.34
49.73
49.70
40.43
39.62
39.25
38.27
35.69
30.86
30.68
29.64
29.44
28.65
27.97
25.45
22.63
21.95
AcOEt
f1 (ppm)

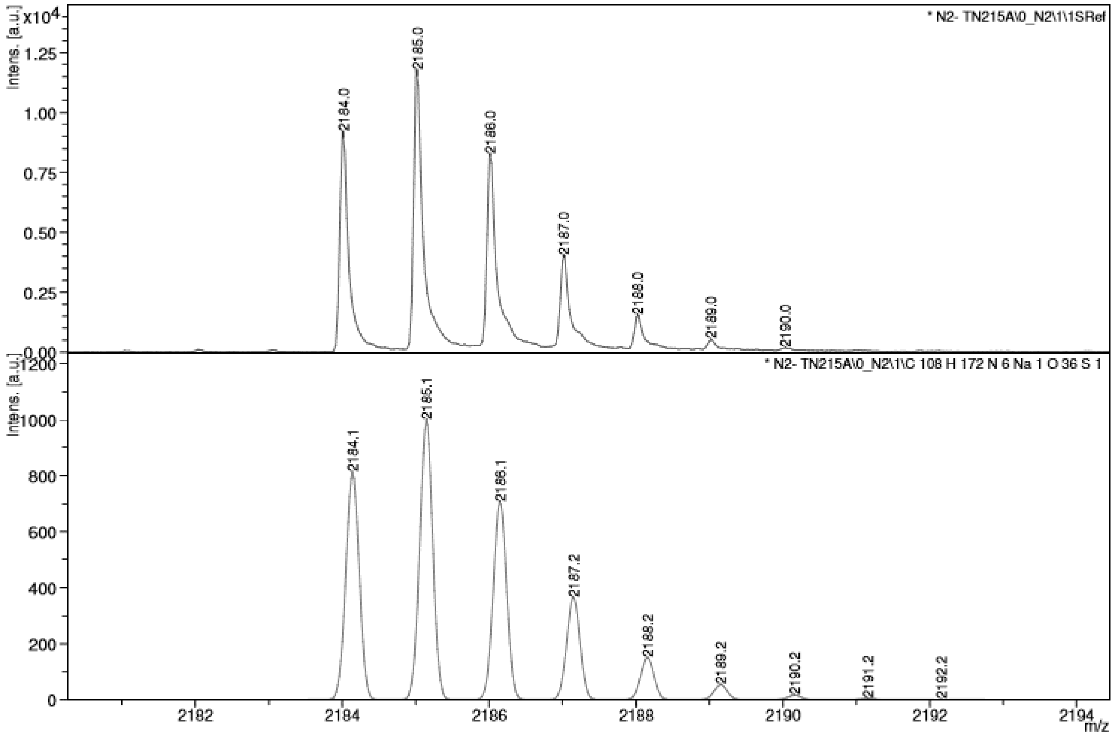
Intens. [a.u.]
x10^4
* N2- TN215A\0_N2\1\1SRef
2184.0
2185.0
2186.0
2187.0
2188.0
2189.0
2190.0
* N2- TN215A\0_N2\1\C 108 H 172 N 6 Na 1 O 36 S 1
2184.1
2185.1
2186.1
2187.2
2188.2
2189.2
2190.2
2191.2
2192.2
m/z

5-azidoisophthaloyl dichloride

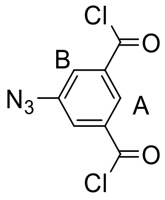

Compound 13

Step i) 5-aminoisophalic acid (2.5 g, 27.6 mmol) was placed in a flash with water (39 mL), 3 mL of 12M HCl was added dropwise. The mixture was cooled in a ice bath at 0ºC. $NaNO_2$ was dissolved in water and added dropwise to the mixture, which was stirred for 30 min. Then, NaN_3 dissolved in water was added dropwise to the mixture. A yellow solid was formed and gas evolution was observed, making it difficult to keep stirring. The mixture was stirred until gas evolution was no longer detected. The product was then filtered, washed with distilled water and dried under vacuum (3.4g, 60%). ^{1}H NMR ([D6]dimethyl sulfoxide): δ = 13.49 (br s, 2H, OH), 8.22 (t, *J* = 1.4 Hz, 1H, Ar-H, H_A), 7.73 (d, *J* = 1.4 Hz, 1H, Ar-H, H_B) ppm.

Step ii) To a stirred suspension of 5-azido-isophthalic acid (100 mg, 0.48 mmol) in DCM (2 mL) two drops of anhydrous DMF and oxalyl chloride (0.15 mL, 1.95 mmol) were added. The reaction mixture was stirred until the product was totally solubilized. The solvent was removed under reduced pressure and the sample was kept 3 h under vacuum to remove oxalyl chloride. The crude product was then used directly in the next reaction step.

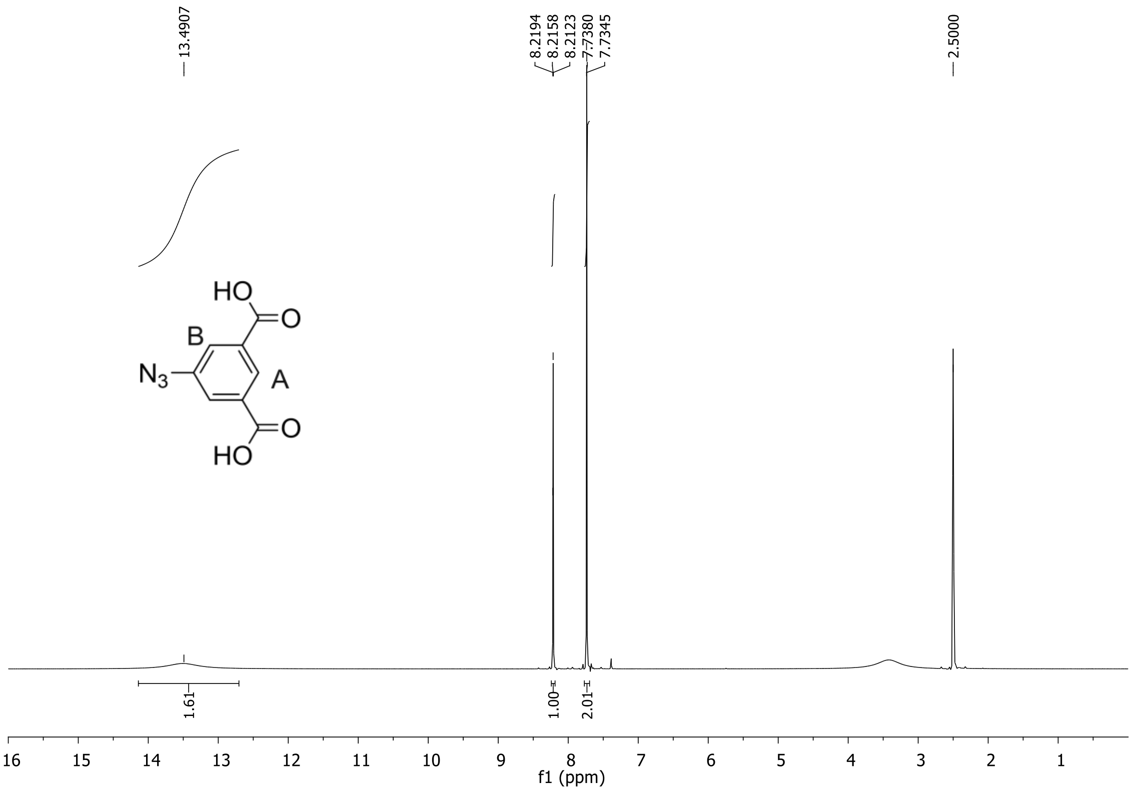

13.4907
8.2194
8.2158
8.2123
7.7380
7.7345
2.5000
HO
O
B
N3
A
O
HO
1.61
1.00
2.01
16
15
14
13
12
11
10
9
8
7
6
5
4
3
2
1
f1 (ppm)

Scheme for the synthesis of compound 14

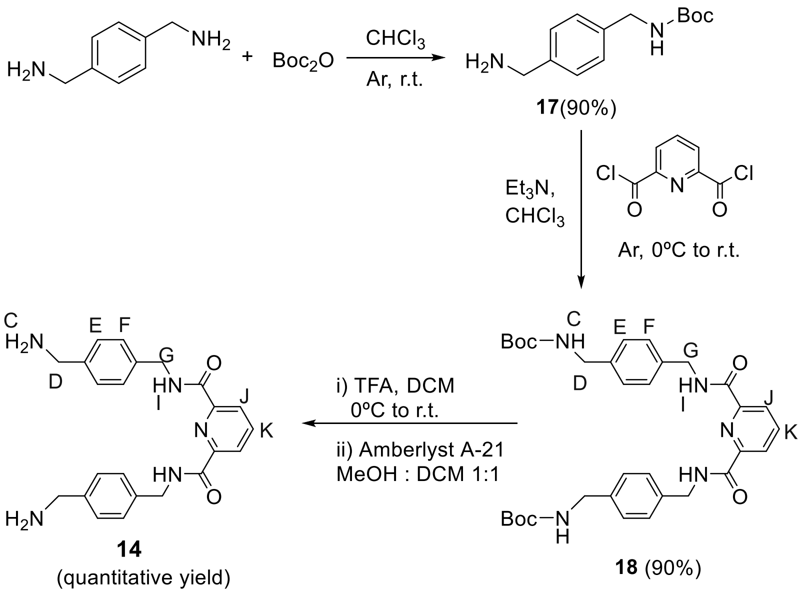

N^2,N^6-bis(4-(aminomethyl)benzyl)pyridine-2,6-dicarboxamide

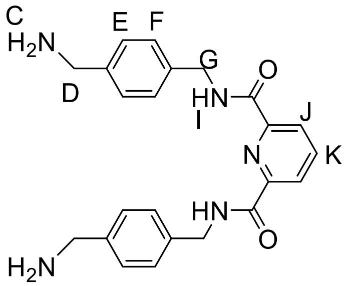

Compound 14

Step i). To a solution of *p*-xylylenediamine (515 mg, 3.78 mmol) in chloroform (60 mL) at 0 ºC, a solution of di-*tert*-butyl carbonate (413 mg, 1.89 mmol) in $CHCl_3$ (45 mL) was added dropwise over a period of 4h. The mixture was stirred at room temperature overnight under Ar. A white solid was filtered from the solution and washed with cold $CHCl_3$. The filtrate was concentrated under reduced pressure. To the remaining oil, DCM and water was added. The layers were separated and the aqueous layer was extracted with DCM (x3). The extracts were combined and dried over Na_2SO_4. The crude material was purified using a gradient elution (DCM/MeOH 15:1

to 6:1) to give compound **17** as yellowish solid (400 mg, 90%) ^{1}H NMR ($CDCl_3$): δ = 7.26 (m, 4H, Ar-H), 4.28 (m, 2H), 3.84 (s, 2H) ppm.

Step ii) 2,6-Pyridinedicarboxylic acid (234 mg, 1.4 mmol) was suspended in anhydrous DCM, the mixture was cooled to 0ºC, then oxalyl chloride was added dropwise followed by the addition of 2 drops of DMF. The reaction mixture was stirred until the product was totally solubilized. The solvent was removed and the crude solid was dried under vacuum for 2 h. The acid chloride (221 mg, 1.1 mmol) dissolved in CH_3Cl, was added dropwise to a solution of compound **14** and Et_3N in CH_3Cl. The reaction mixture was stirred overnight. The solvent was removed under reduced pressure. To the remaining oil, DCM and water were added. The layers were separated and the aqueous layer was extracted with DCM (x3). The extracts were combined and dried over Na_2SO_4. The crude material was purified using column chromatography, with a gradient elution (DCM/MeOH 15:1 to 6:1) to give compound **18** as yellowish solid (400 mg, 90%) ^{1}H NMR ($CDCl_3$): δ = 8.90 (br s, 2H, NH, H_I), 8.27 (d, *J* = 7.8 Hz, 2H, Ar-H, H_J), 7.95 (t, *J* = 7.8 Hz, 1H, Ar-H, H_K), 7.03 (m, 4H, H_F), 6.97 (m, 4H, H_E), 5.28 (br s, 2H, NH, H_C), 4.31 (br d, *J* = 5.5 Hz, 4H, H_G), 4.09 (br d, *J* = 5.9 Hz, 4H, H_D), 1.40 (s, 18H, Boc-H); ^{13}C NMR ($CDCl_3$): δ = 163.8, 156.2, 148.8, 138.7, 138.1, 137.2, 127.7, 127.2, 125.0, 79.5, 44.1, 43.0, 28.4 ppm.

Step iii) Compound **18** was dissolved in DCM, the solution was cooled to 0 ºC and TFA was added dropwise until the reaction was completed (TLC). Solvent was removed under vacuum. The crude material was dissolved in DCM/MeOH 1/1 v/v ratio and was stirred with Amberlyst A-21 resin for 1h. The solvent was removed under reduced pressure to yield compound **14** as a hygroscopic powder (quantitative yield). ^{1}H NMR ([D6]dimethyl sulfoxide): δ = 9.92 (br t, *J* = 6.4 Hz, 2H, NH, H_I), 8.22 (m, 3H, Ar-H, H_J, H_K), 7.37 (m, 4H, H_F), 7.33 (m, 4H, H_E), 4.31 (br d, *J* = 6.4 Hz, 4H, H_G), 4.09 (br s, 4H, H_D).

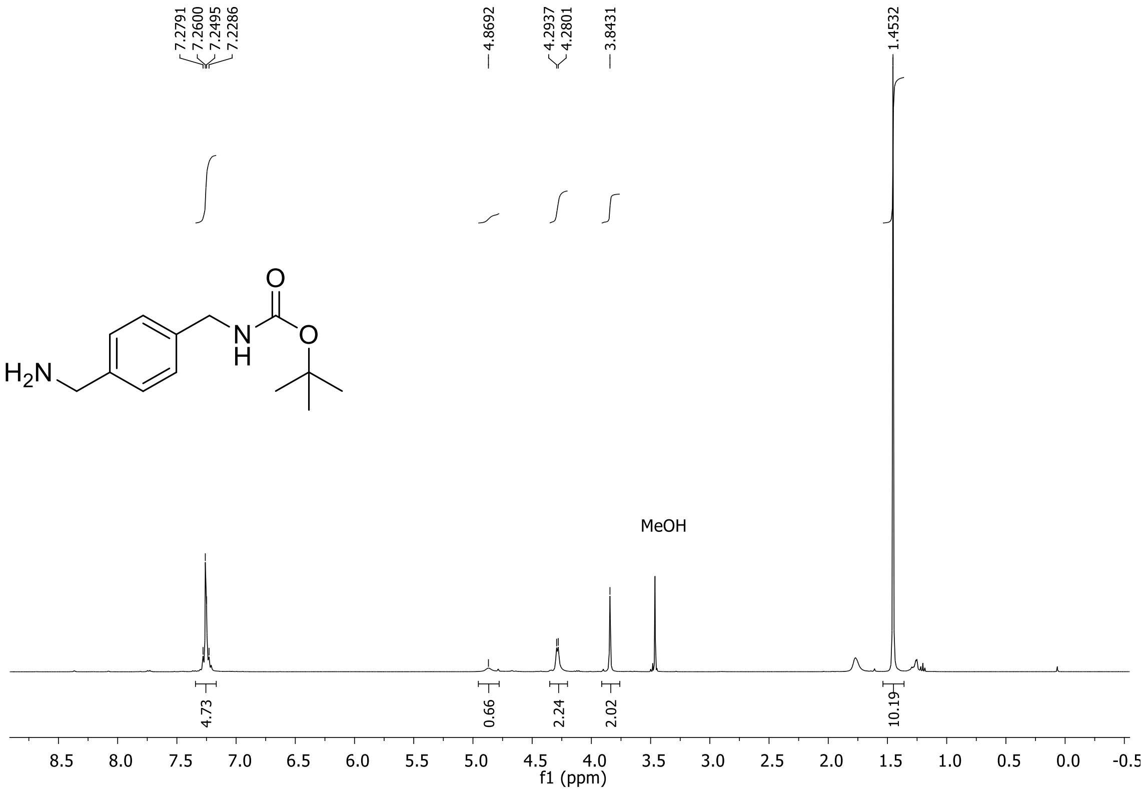

7.2791
7.2600
7.2495
7.2286
4.8692
4.2937
4.2801
3.8431
1.4532
H2N
N
H
O
O
MeOH
4.73
0.66
2.24
2.02
10.19
8.5 8.0 7.5 7.0 6.5 6.0 5.5 5.0 4.5 4.0 3.5 3.0 2.5 2.0 1.5 1.0 0.5 0.0 -0.5
f1 (ppm)

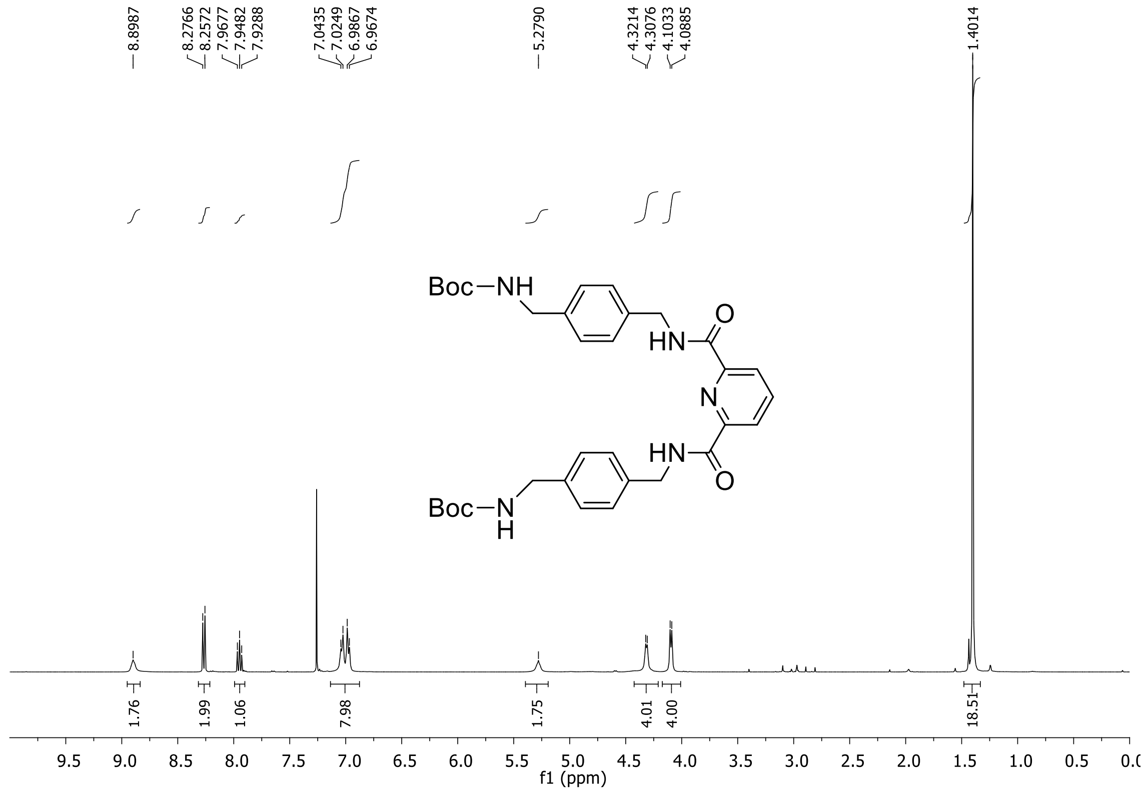

8.8987
8.2766
8.2572
7.9677
7.9482
7.9288
7.0435
7.0249
6.9867
6.9674
5.2790
4.3214
4.3076
4.1033
4.0885
1.4014
Boc—NH
HN
O
N
HN
O
Boc—N
H
1.76
1.99
1.06
7.98
1.75
4.01
4.00
18.51
9.5 9.0 8.5 8.0 7.5 7.0 6.5 6.0 5.5 5.0 4.5 4.0 3.5 3.0 2.5 2.0 1.5 1.0 0.5 0.0
f1 (ppm)

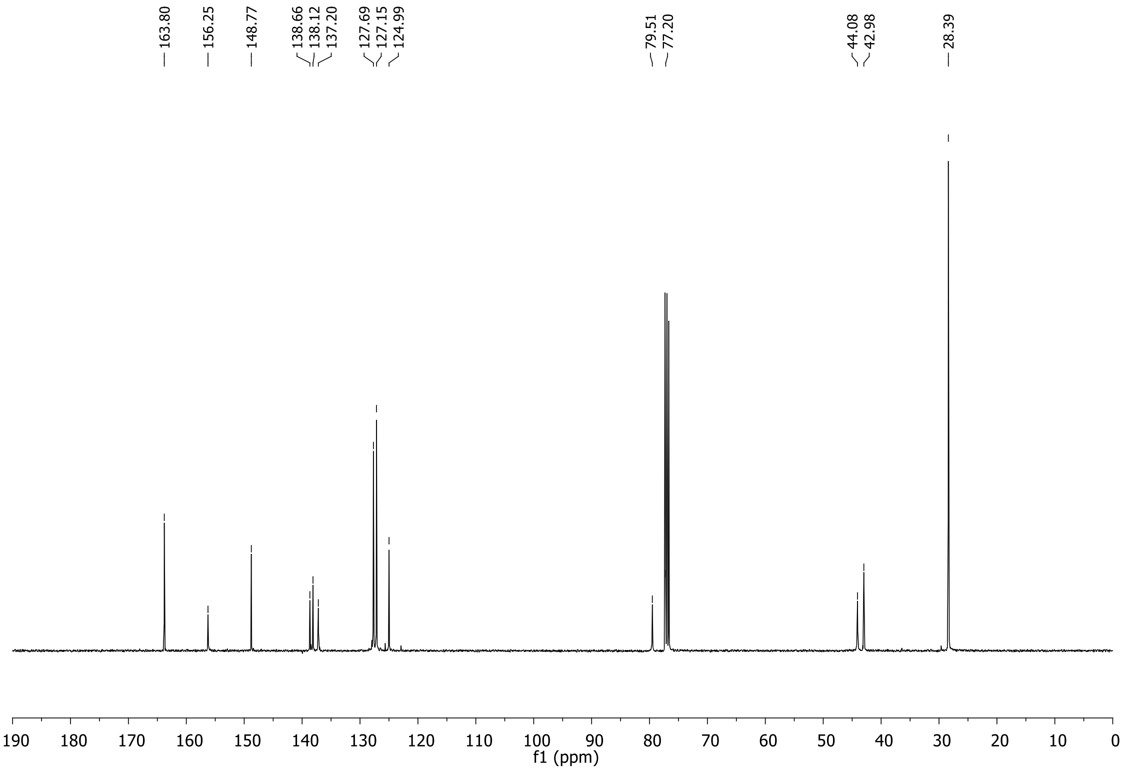
163.80
156.25
148.77
138.66
138.12
137.20
127.69
127.15
124.99
79.51
77.20
44.08
42.98
28.39
190 180 170 160 150 140 130 120 110 100 90 80 70 60 50 40 30 20 10 0
f1 (ppm)

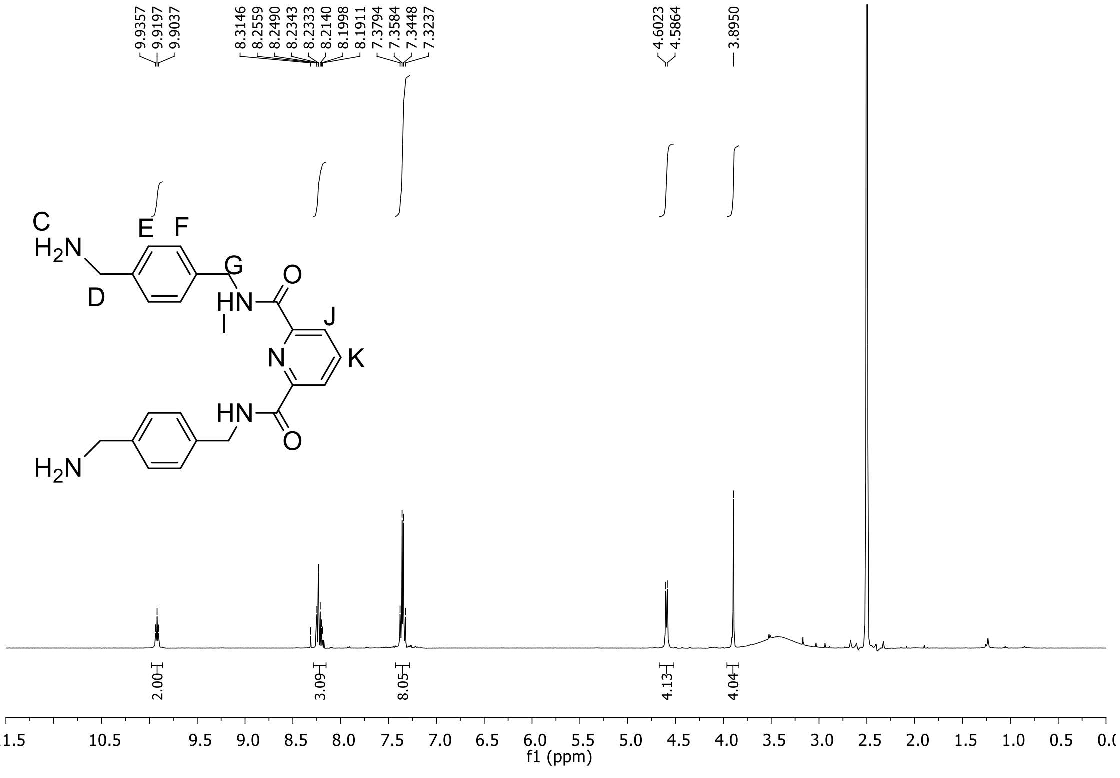
9.9357
9.9197
9.9037
8.3146
8.2559
8.2490
8.2343
8.2333
8.2140
8.1998
8.1911
7.3794
7.3584
7.3448
7.3237
4.6023
4.5864
3.8950
C
E F
G
D
I
J
K
2.00
3.09
8.05
4.13
4.04
11.5 10.5 9.5 9.0 8.5 8.0 7.5 7.0 6.5 6.0 5.5 5.0 4.5 4.0 3.5 3.0 2.5 2.0 1.5 1.0 0.5
f1 (ppm)

[2]Rotaxane

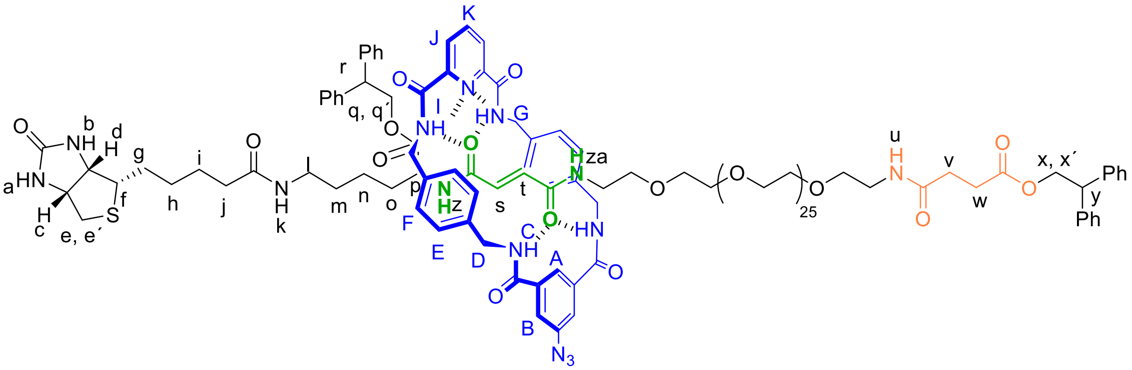

Compound 15

Thread **12** (16 mg, 7.3 x 10^{-3} mmol), was dissolved in 1.5 mL $CHCl_3$ (stabilized with amylenes), Et_3N (30 μL, 0.22 mmol) was added and the reaction mixture were stirred vigorously while solutions of the diamine, compound **14**, (36 mg, 0.09 mmol) in $CHCl_3$ (0.7 mL) and the acid chloride, compound **13** (21 mg, 0.09 mmol) in $CHCl_3$ (0.7 mL) were simultaneously added over a period of 3h using motor-driven syringe pumps. After 24h the resulting suspension was filtered and the solvent removed under reduced pressure. The crude material was purified by column chromatography (gradient elution: DCM/ MeOH 25:1 to 18:1) to furnish rotaxane **15** as a colorless oil (7 mg, 35%); ^{1}H NMR ([D6]dimethyl sulfoxide): δ = 9.83 (m, 1H, NH), 9.56 (m, 2H, NH), 9.18 M (m, 1H, NH), 8.72 (d, *J* = 7.1 Hz, 1H, NH_t or NH_z), 8.52 (t, *J* = 5.7 Hz, 1H, NH_z or NH_{za}), 8.31 (m, 1H, Ar-H), 8.22 (m, 2H, Ar-H), 8.11 (m, 1H, NH), 7.82 (m, 1H, NH), 7.70 (m, 1H, NH), 7.63 (m, 2H, Ar-H), 7.28 (m, 16 H, Ar-H), 7.21 (m, 8H, Ar-H), 7.13 (m, 4 H, Ar-H), 6.51 (m, 2H, H_s, H_t), 6.41 (br s, 1 H, H_a), 6.35 (br s, 1 H, H_b), 4.72 (dd, *J* = 8.1, 10.9 Hz, 1 H, H_q or $H_{q'}$), 4.56 (m, 7H, $H_{q'}$ or H_q, H_x, $H_{x'}$ and H_D or H_G), 4.37 (m, 7H, H_c, H_y, H_r and H_D or H_G), 4.11 (m, 2H, H_d, H_p), 3.50 (m, 14H, EG26-H), 3.40 (m, 90H, EG26-H), 3.17 (m, 2H, EG26-H), 3.08 (m, 1H, H_f), 2.91 (m, 2 H, H_l), 2.81 (m, 1 H, H_e or $H_{e'}$), 2.54 (d, *J* = 12.8 Hz, 1 H, $H_{e'}$ or H_e), 2.32 (m, 2 H, H_w), 2.21 (t, *J* = 6.6 Hz, 2 H, H_v), 2.03 (t, *J* = 6.8 Hz, 2 H, H_j), 1.44 (m, 12 H, H_g, H_i, H_h, H_n, H_m, H_o) MS m/z: calculated for $C_{108}H_{172}N_6O_{36}S$ $[M+Na]^+$ 2759.4 found MALDI-TOF 2763.3.

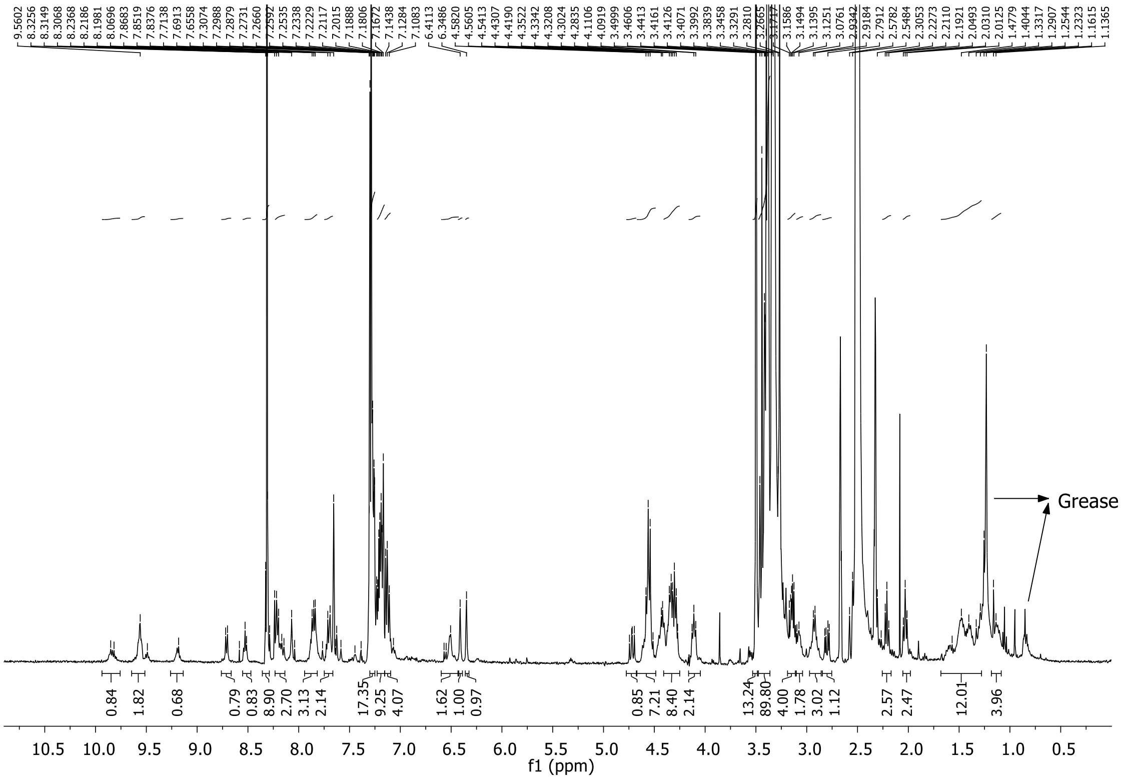
Grease
0.84
1.82
0.68
0.79
0.83
8.90
2.70
3.13
2.14
17.35
9.25
4.07
1.62
1.00
0.97
0.85
7.21
8.40
2.14
13.24
89.80
4.00
1.78
3.02
1.12
2.57
2.47
12.01
3.96
10.5 10.0 9.5 9.0 8.5 8.0 7.5 7.0 6.5 6.0 5.5 5.0 4.5 4.0 3.5 3.0 2.5 2.0 1.5 1.0 0.5
f1 (ppm)

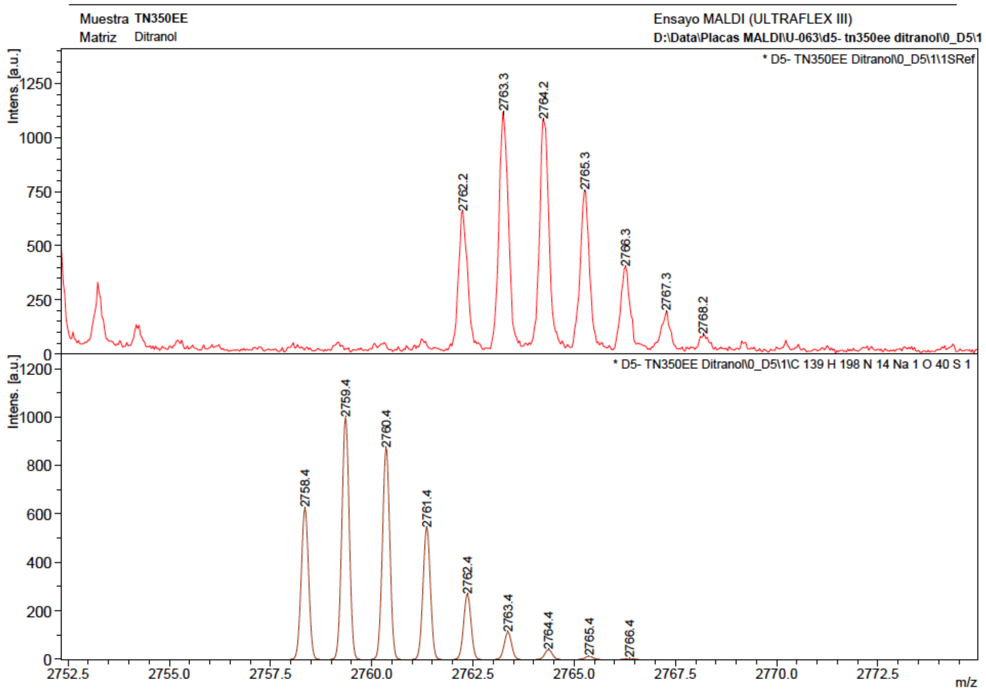
Muestra TN350EE
Matriz Ditranol
Ensayo MALDI (ULTRAFLEX III)
D:\Data\Placas MALDI\U-063\d5- tn350ee ditranol\0_D5\1
* D5- TN350EE Ditranol\0_D5\1\1SRef
Intens. [a.u.]
2762.2
2763.3
2764.2
2765.3
2766.3
2767.3
2768.2
* D5- TN350EE Ditranol\0_D5\1\C 139 H 198 N 14 Na 1 O 40 S 1
2758.4
2759.4
2760.4
2761.4
2762.4
2763.4
2764.4
2765.4
2766.4
2752.5 2755.0 2757.5 2760.0 2762.5 2765.0 2767.5 2770.0 2772.5
m/z

Position of the macrocycle in solution

Comparison of the ^{1}H NMR spectra of axle, compound **12**, and [2]rotaxane compound **15** (depicted in the next two figures) in [D6]dimethyl sulfoxide (400 MHz, 298K) indicates that the macrocycle predominantly resides over the fumaramide station. The H_s and H_t protons of the fumaramide group are strongly shielded in the rotaxane compared to the axle evidenced by a shift in their peak positions by 0.34 ppm, whereas the chemical shifts of the H_v and H_w protons of the succinic amide-ester group are only slightly shifted by 0.05 ppm.

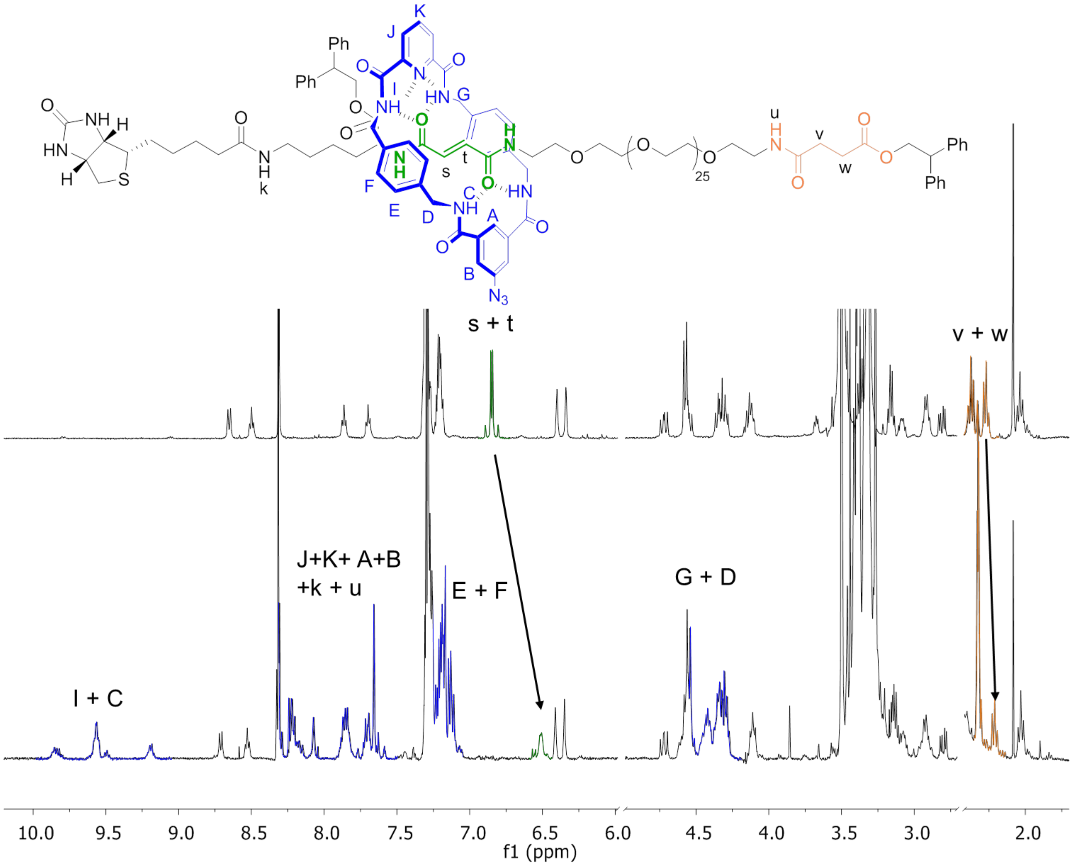

The figure above compares compound **12**, (*E*)-thread, (top spectrum) and compound **15**, rotaxane molecule, (bottom spectrum) Showing in green the protons corresponding to the fumaramide double bond, which are shifted in 0.34 ppm in the rotaxane molecule; in orange we can find the protons of the succinic-amide ester group, which are shifted only by 0.05 ppm in the rotaxane molecule. Finally we can find the protons corresponding to the macrocycle highlighted in blue.

S2 Supplementary figures

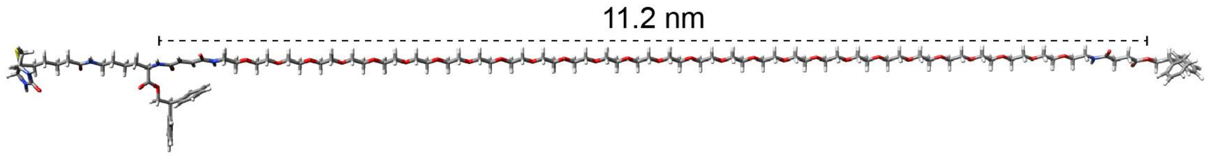

Supplementary figure 1: Distance between stations in the gas phase. Energy-minimized (MMF94s) structure of the axle, marking the distance (11.2 nm) between the N atom of the *fum* station and the O atom of the *succ* station.

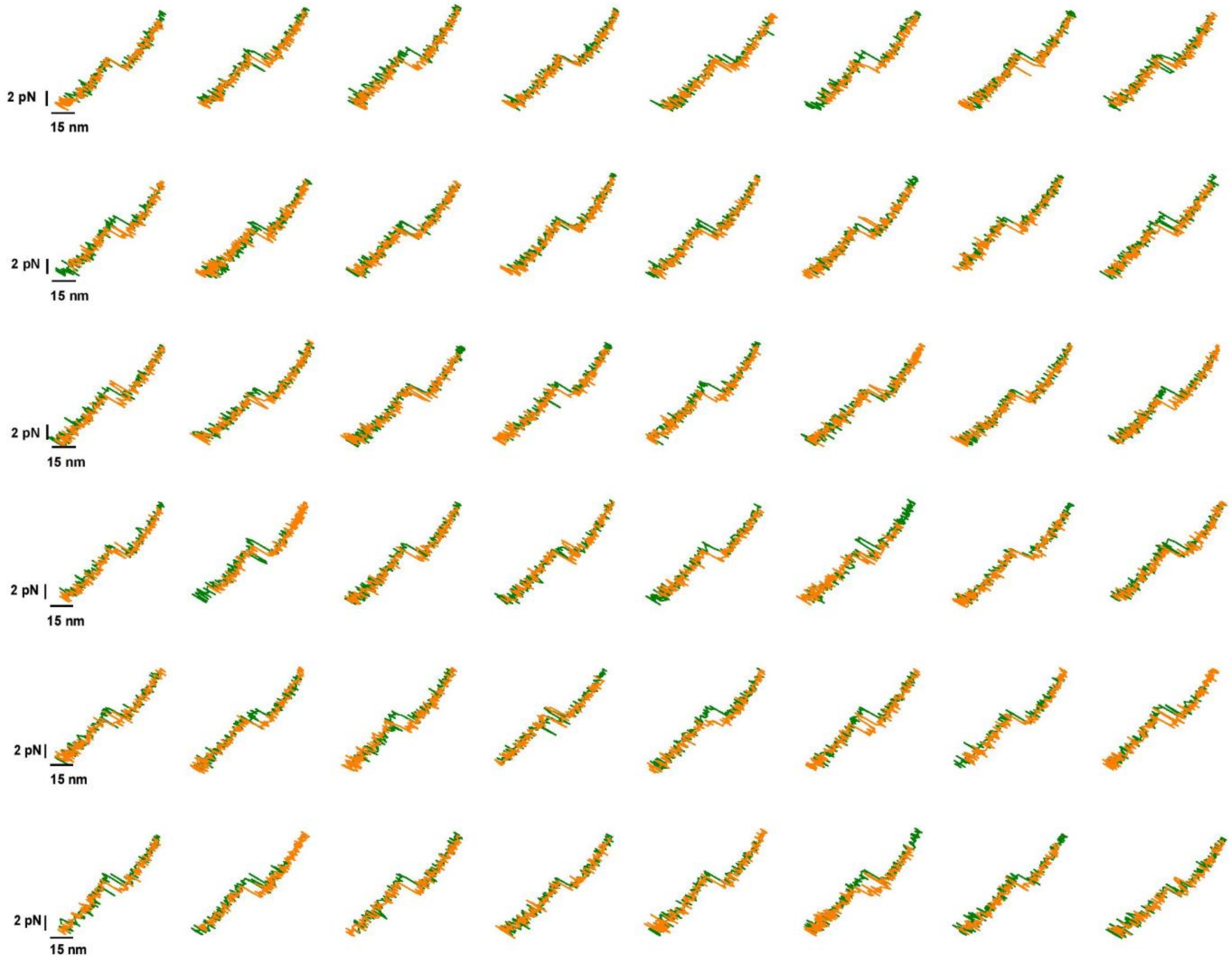

Supplementary figure 2: Consecutive force-extension curves of a single rotaxane. The figure shows 48 consecutive pull (green)-relax (orange) cycles of a representative rotaxane-DNA construct (pulling rate 200 nm s^{-1}). Each pull-relax cycle is shown in an independent plot for clarity of display. The robustness of our method allowed us to obtain up to 120 pull-relax curves from a single molecule. The bi-stability of the molecule can be seen clearly in several curves as rapid oscillations of distance at forces close to $f_{1/2}$.

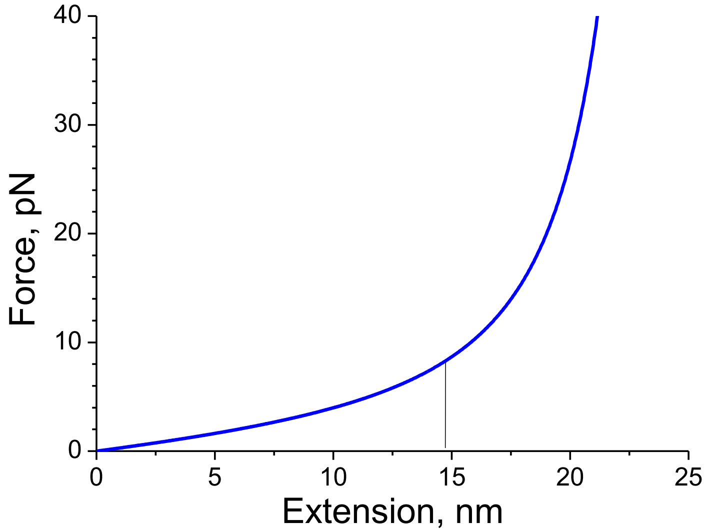

Supplementary figure 3: Determination of the free energy of stretching of the rotaxane-DNA hybrid. The force-extension curve of the rotaxane-DNA hybrid after shuttling, when the macrocycle is at the *succ* station, results from the elastic properties of the dsDNA (spacer and handle, 3615 bp) and those of the rotaxane. The Worm-Like-Chain model (WLC) of polymer elasticity is known to predict well both, the extension of dsDNA[23] and the extension of a linear polymer, like PEG[29], as a function of the applied force. In fact, the force-extension curve of the rotaxane-DNA hybrid after shuttling is well fit by an expression including the sum of two WLC models; one for the dsDNA, with persistence and contour lengths of L_p = 50 nm and L_c = 0.34 nm bp^{-1}, respectively[23], and another for the rotaxane, with L_p = 0.9 ± 0.2 nm and L_c = 21.6 ± 1 nm, under our experimental conditions. The figure shows the force-extension curve of the rotaxane according to a WLC model including the L_p and L_c values determined from the fits. As expected, a length of 14.96 nm (indicated by a line) for the fully extended rotaxane at the coexistence force (8.61 pN) is predicted, which is fully compatible with the shuttling distance measured experimentally, ~15 nm. The free energy of stretching of the rotaxane ($\Delta G_{stretch}$) was calculated by integrating the area under the curve from 0 pN to the coexistence force, $\Delta G_{stretch}$= 11.8 k_BT.

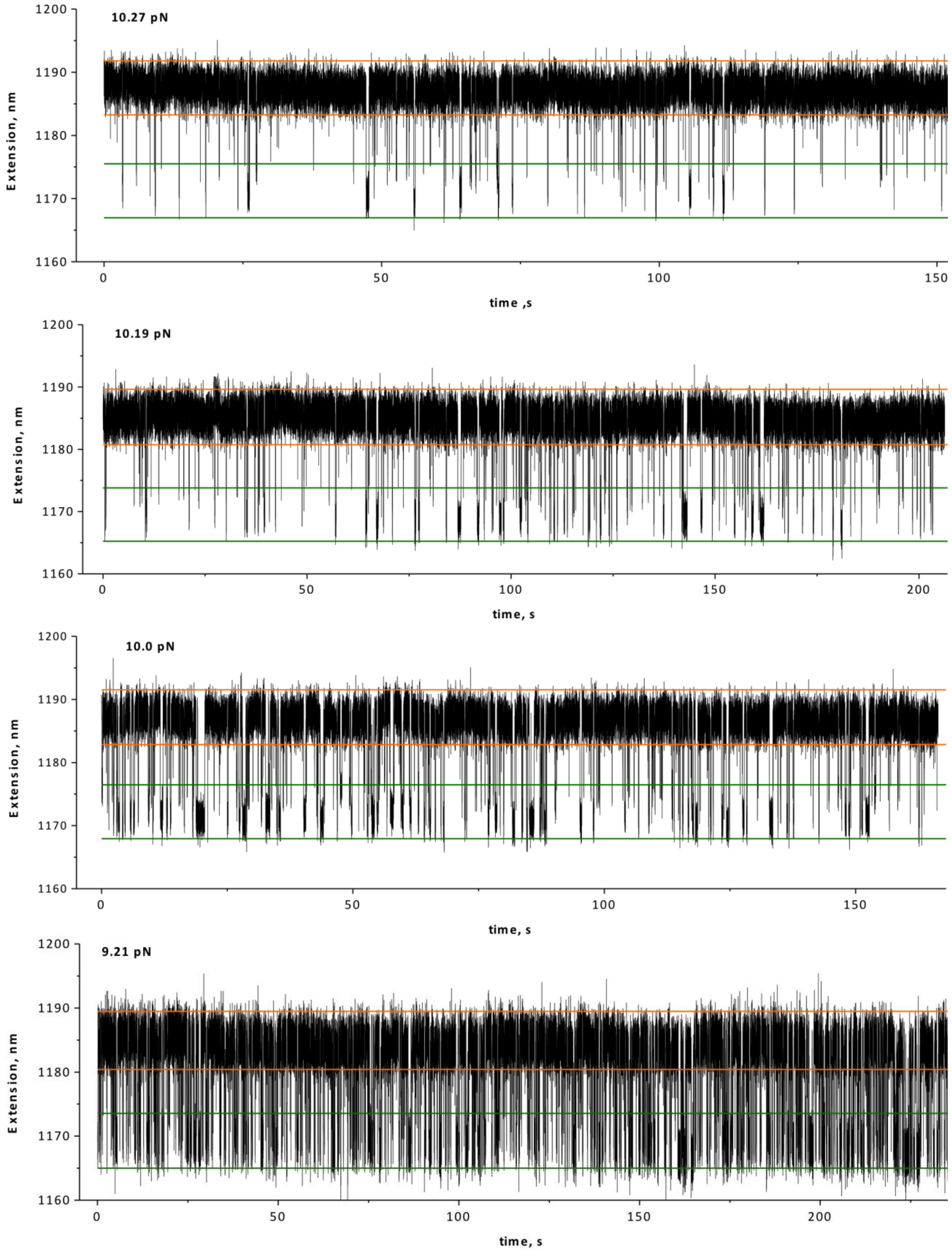

10.27 pN
Extension, nm
time ,s
10.19 pN
Extension, nm
time, s
10.0 pN
Extension, nm
time, s
9.21 pN
Extension, nm
time, s

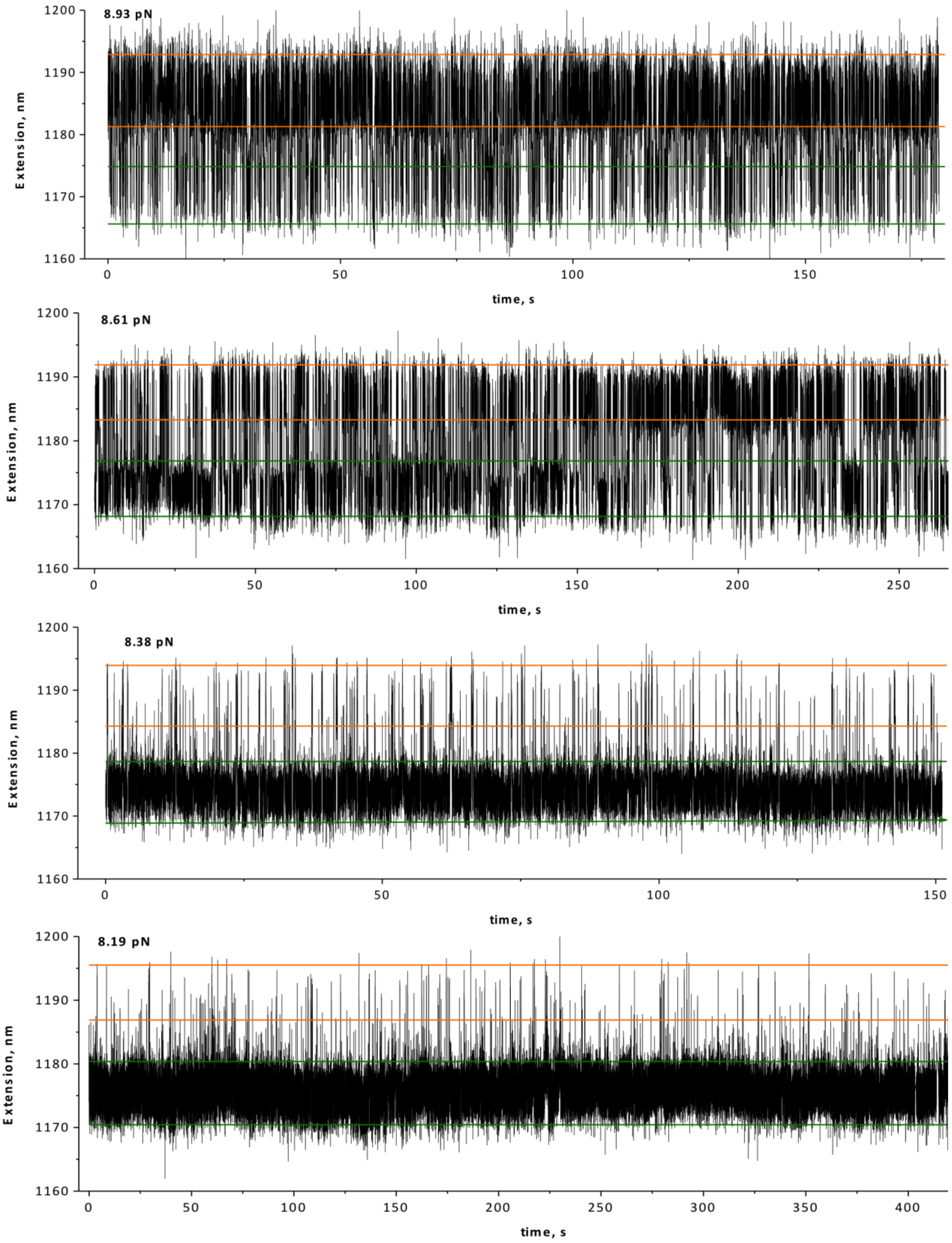

Supplementary figure 4: Examples of the shuttling dynamics for independent rotaxane-DNA constructs at different constant forces. Green and orange lines represent the extension of the DNA handles when the *fum* and *succ* stations, respectively, are active.

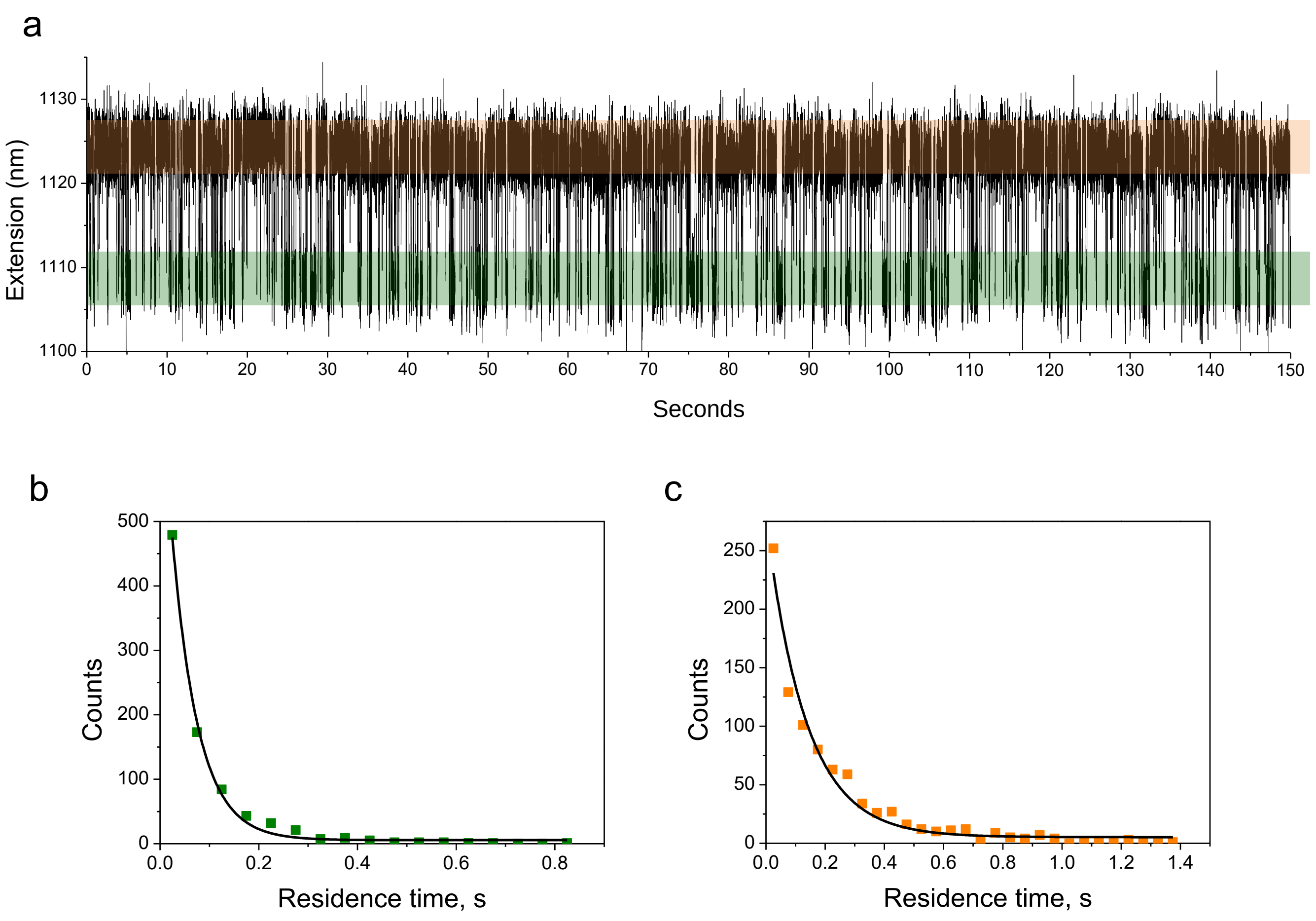

Supplementary figure 5: Distributions of residence times. **a** Real-time extension changes vs. time of a molecular shuttle hold at a constant force of 9.2 pN. For clarity of display only the first 150 seconds are shown (total recording time for this molecular shuttle was 240 s). Green and orange boxes represent the extension of the dsDNA handles when the *fum* and *succ* stations, respectively, are active. **b** The residence time distribution at the *fum* station for molecule shown in (A) fits well to a single exponential (black line). **c** Residence time distribution at the *succ* station for molecule shown in (A) fitted to a single exponential (black line). As expected at force higher that $f_{1/2}$ the macrocycle spends longer times at the *succ* station.

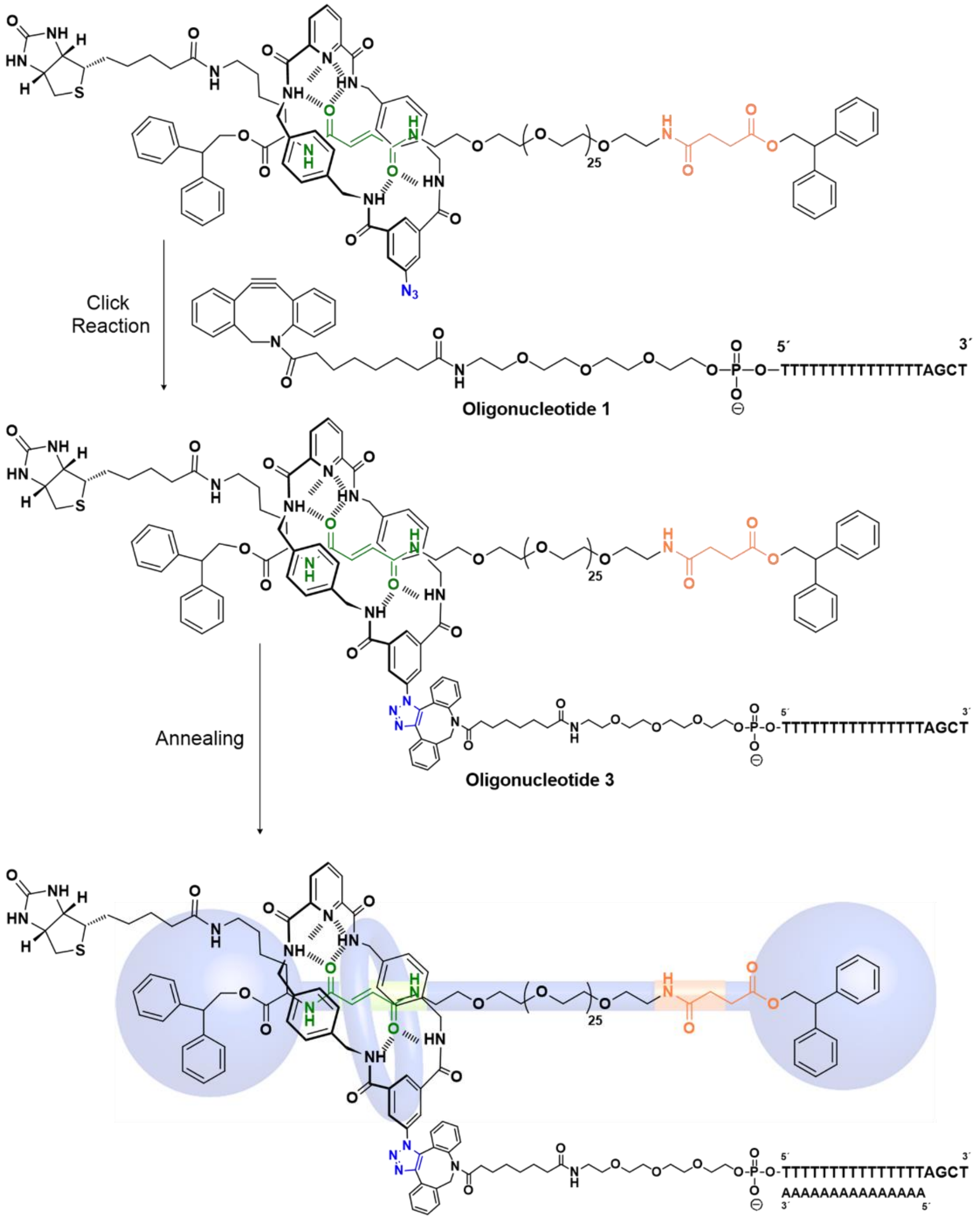

Supplementary figure 6: Scheme of the copper-free "click" reaction for the initial step of coupling of the macrocycle with dsDNA.
